# Calcium Homeostasis Is Involved in the Modulation of Gene Expression by MSL2 in Imbalanced Genomes

**DOI:** 10.3390/cells13221923

**Published:** 2024-11-20

**Authors:** Ruixue Wang, Shuai Zhang, Haizhu Qi, Liuqing Wang, Youjun Wang, Lin Sun

**Affiliations:** 1 Key Laboratory of Cell Proliferation and Regulatory Biology, Ministry of Education, College of Life Sciences, Beijing Normal University, Beijing 100875, China; 2 Beijing Key Laboratory of Gene Resource and Molecular Development, College of Life Sciences, Beijing Normal University, Beijing 100875, China

**Keywords:** aneuploidy, genomic imbalance, MSL2, *ERp60*, calcium homeostasis

## Abstract

Aneuploidy is highly detrimental to organisms due to genomic imbalance. However, the influence of parental unbalanced genome conditions on gene expression of their offspring remains unclear, particularly in animals. To further explore the molecular regulatory mechanisms, we firstly analyzed the expression patterns of aneuploid *Drosophila* offspring from different parents with unbalanced genomes via reciprocal crosses and studied the potential functions of male-specific lethal 2 (MSL2) in this process. The results showed that the ectopic expression of MSL2 in aneuploidy resulted in gene expression patterns closer to those of diploidy, including MSL2 target genes, maternal genes, mitochondrial genes, and transposable elements. In addition, it was also found that *ERp60*, the key target gene of MSL2, played a crucial role in regulating endoplasmic reticulum (ER) Ca^2+^ homeostasis through its interaction with the STIM1 protein. When it was overexpressed, ER Ca^2+^ levels and the survival of aneuploid females were significantly increased. Furthermore, we observed upregulated ER Ca^2+^ levels identified in aneuploid brains, which suggested that Ca^2+^ homeostasis may be involved in the regulation mediated by MSL2 in aneuploid genomes.

## 1. Introduction

Aneuploidy, characterized by an abnormal number of chromosomes that are not a multiple of the haploid set, results in genomic imbalance [1]. Studies have indicated that altering the dosage of specific genomic segments often has more pronounced effects on an organism than changes in the entire genome [2]. In humans, aneuploidy is a leading cause of congenital miscarriages, with only a minority of aneuploid conditions being viable for live births [3,4]. Aneuploidy can also cause growth retardation, developmental defects, and mortality in other species, such as mice [5], *Datura* [6], maize [7], and *Arabidopsis* [8]. Furthermore, aneuploidy serves as a hallmark of cancer, observed in approximately 91% of glioblastomas and 65% of lung tumors [9]. In the process of constructing aneuploidy by using the model organism *Drosophila*, we found that the offspring generated by changing the parents who provide the imbalanced gametes (i.e., reciprocal crosses) have slightly different phenotypes, although their genotypes are the same, which are called reciprocal effects [10,11]. However, the phenotypic effects and molecular regulatory mechanisms of parental genomic imbalance on the offspring remain to be elucidated.

In response to the genomic imbalance that occurs naturally in male *Drosophila*, the male-specific lethal (MSL) complex has evolved to recruit regulators, such as the histone acetylase male absent on the first (MOF), from the autosomes to the X chromosome [12]. MSL2 is responsible for the assembly of the MSL complex [13,14]. Previous studies have demonstrated an interaction between the MSL complex and genomic imbalance through MSL2 overexpression. In addition, MSL2 is also essential for maintaining biallelic transcription for some genes by sustaining the connection between promoters and enhancers [15]. Previous studies have found a sexual dimorphism in unbalanced genomes and more notable effects on females than males after MSL2 protein overexpression [16,17]. This study focused on the functions of MSL2 under the condition of parental genomic imbalance and analyzed some relevant factors that may lead to the reciprocal effects.

Reciprocal effects have been reported in different species, such as maize, tomato, *Arabidopsis thaliana*, and *Drosophila* [18,19,20,21]. Typically, these phenotypic differences may be associated with various mechanisms, such as maternal effects and cytoplasmic inheritance [10,22]. These mechanisms are all related to the differential contribution of maternal and paternal parents to the offspring, known as the parent-of-origin effects [23], which might play an important role in genetic imbalance. Maternal effects refer to the influence of the maternal genotype or phenotype on the phenotype of the offspring. After fertilization, the normal development of early embryos before zygotic genome activation (ZGA) needs a maternal parent to provide nutrients, transcripts, hormones, and signaling molecules [24]. Cytoplasmic inheritance is caused by an asymmetrical distribution of mitochondria and plastids during gametogenesis or fertilization [25].

Other types of regulators that may be implicated in reciprocal crosses are transposable elements (TEs), also known as mobile genetic elements. For example, the P element is known to cause “P-M hybrid dysgenesis” in *Drosophila* [26]. In addition, the evolution of high-affinity chromatin entry sites (CESs) of different X chromosomes in *D. miranda* illustrates that Helitron transposons play an important role in the localization of the MSL complex, indicating a correlation between TEs and sex chromosome evolution [27,28].

The key gene *ERp60*, potentially influenced by MSL2 under conditions of genomic imbalance, is associated with the endoplasmic reticulum (ER), the primary reservoir of calcium ions (Ca^2+^) in cells. Due to the spatiotemporal specificity of calcium signaling in cells, Ca^2+^ can mediate various physiological processes, including sperm–egg binding, cell adhesion, gene transcription, protein translation, and cell apoptosis [29]. A recent study demonstrated that the maternal effect factor NLRP14 maintains Ca^2+^ oscillations and Ca^2+^ homeostasis, ensuring early embryonic development [30]. Abnormal resting Ca^2+^ levels, known as dysregulated Ca^2+^ homeostasis [31], can lead to various diseases such as cardiovascular diseases, immunodeficiency, neurodegenerative diseases, and cancer [32,33]. In addition, such imbalances have been observed in aneuploidy. For example, multiple types of neurons cultured from trisomy 16 fetal mice (Ts16) reportedly showed changes in the amplitude of voltage-dependent Ca^2+^ currents or activation kinetics for Ca^2+^ currents [34,35].

In this study, we found that aneuploidies disrupted global gene expression and led to significant phenotypic effects. Specifically, we observed a decrease in the number of female offspring and an elevation in ER Ca^2+^ levels within the brain. Our further investigation into the overexpression of MSL2 in trisomy 2L showed that the skewed sex ratios and altered gene expression profiles could be partially corrected by inducing MSL2 overexpression. This suggested a potential regulatory role of MSL2 in mitigating the effect of parental genomic imbalance. Importantly, we discovered that *ERp60*, a gene that may be influenced by MSL2, plays a critical role in maintaining Ca^2+^ homeostasis in the ER, depending on the stromal interaction molecule 1 (STIM1) protein. STIM is a transmembrane protein on the ER membrane, which together with the Orai protein in the plasma membrane (PM) mediates the store-operated calcium entry (SOCE). These findings underscore the intricate interplay between genetic imbalance, gene expression regulation, and Ca^2+^ homeostasis in aneuploidy.

## 2. Methods

### 2.1. Drosophila Stocks and Crosses

The crossing method is shown in Figure S1. Trisomy chromosome 2 left arm (2L) larval samples were obtained from crosses of *y;C(2L) dp*; *F(2R) bw* females with Canton S males and Canton S females with *y;C(2L) dp*; *F(2R) bw* males, which were denoted as trisomy 2L+ and trisomy 2L-, respectively (Figure S1A,B). Larval samples of trisomy 2L with ectopic MSL2 expression were obtained from the cross combinations of *y;C(2L) dp*; *F(2R) bw* females with *MSL2^OE^/MSL2^OE^* males and *MSL2^OE^/MSL2^OE^* females with *y;C(2L) dp*; *F(2R) bw* males, denoted as MSL2-trisomy 2L+ and MSL2-trisomy 2L- (Figure S1C,D). Strains with homozygous MSL2 transgenes on chromosome 2R were constructed previously [16].

To record the number and sex ratio of reciprocal F1 hybrids, we chose the same number and age of parents to cross. The parents were removed after four days. The number of F1 surviving male and female third instar larvae in each hybrid was recorded three times a day. We distinguish the sex of larvae by observing the presence of testes. The testes of male larvae are two bilateral oval translucent structures, located in the posterior third of the body. The statistical results of three hybridizations were regarded as one biological replication, and more than three biological replications were set.

### 2.2. Salivary Gland Chromosome Immunostaining

Salivary glands from third instar larvae were dissected. Then, they were fixed and dissociated in 3.7% formaldehyde (Beijing Chemical Plant, Beijing, China) and a mixture of 50% acetic acid (Xilong Chemical Co., Ltd. Shanghai, China) and 3.7% formaldehyde. The primary antibodies used were anti-SXL (M18-s, Developmental Studies Hybridoma Bank, Iowa City, IA, USA) and anti-MSL2 (sc-32459, Santa Cruz, Dallas, TX, USA) at a dilution of 1:100, and the conjugated secondary antibodies used were Alexa Fluor 488 (705-545-147, Jackson ImmunoResearch, West Grove, PA, USA) and Alexa Fluor 594 (715-585-150, Jackson ImmunoResearch) at a 1:200 dilution [36].

### 2.3. Embryo Immunostaining

To collect embryos, 100 male and 200 female flies were used to lay eggs in cages, each of which was covered by a Petri dish with an agar juice medium (Aobox, Beijing, China).

For H4K16Ac staining, embryos were fixed with methanol/heptane (1:1) and washed with methanol (Modern Oriental Technology Development Co., Ltd., Beijing, China) three times [37]. The methanol and heptane (Tokyo Chemical Industry, Shanghai, China) were precooled at 4 °C. Subsequently, the embryos were washed with a mixture of PBS, 0.1% Tween-20 (Amresco VWR, Shanghai, China), and 0.3% Triton-X 100 (Sigma, Shanghai, China) (PBTT) and blocked with 0.5% BSA (Solarbio, Beijing, China) before they were incubated for 2 h with anti-H4K16Ac antibodies (07-329, EMD Millipore, Billerica, MA, USA) at room temperature. After washing with PBTT, the embryos were incubated with 4′,6-diamidino-2-phenylindole (DAPI) (D9542, Sigma, Shanghai, China) and a secondary antibody (705-545-147, Jackson ImmunoResearch) for 15 min and 2 h at room temperature, respectively. The embryos were subsequently washed three times with PBTT and PBS [38]. Microscopy imaging was performed using a LSM 880 laser confocal fluorescence microscope (Zeiss, Shanghai, China).

### 2.4. Embryo RNA Extraction and Relative Quantitative PCR

For RNA extraction, embryos were collected for 30 min on apple juice agar plates and aged at 25 °C to reach the desired stage, pre-maternal-to-zygotic transition (MZT) and pre-ZGA for 15 min, post-ZGA for 2.5 h, and post-MZT for 5.5 h [39]. RNA was isolated using the TRIzol reagent (Invitrogen, Carlsbad, CA, USA) from the embryos at the indicated stages, and cDNA was generated with TransScript One-Step gDNA Removal and cDNA Synthesis SuperMix (TransGen Biotech, Beijing, China) [28]. Real-time PCR was performed with the diluted cDNA and the TransStart Tip Green qPCR SuperMix (TransGen Biotech) for at least three biological and technical repeats. The primers used for RNA quantification are listed in Table S1. The results were analyzed and plotted using the QuantStudio real-time PCR software v1.3 (Applied Biosystems, Foster City, CA, USA) and GraphPad Prism 7 (GraphPad Software, Boston, MA, USA).

### 2.5. Plasmid Construction

To generate pcDNA3.1-mScarlet, the sequence of mScarlet was first amplified by PCR from PEB2-mScarlet (pEB2-mScarlet was a gift from Philippe Cluzel (Harvard University), Addgene plasmid # 104006; RRID: Addgene_104006, Watertown, MA, USA) [40] and then subcloned into the pcDNA3.1(+) backbone by using a multiple fragment homologous recombination kit (C113, Vazyme Biotech, Nanjing, China). For pcDNA3,1-mScarlet-ERP60, the signal peptide-encoding sequence and the remaining nucleotides were inserted upstream and downstream of the mScarlet gene, respectively. The sequence of *ERP60* was synthesized by Qinglan Biotech (Shenzhen, China).

### 2.6. Cell Culture and Transfection

All cells were cultured in Dulbecco’s modified Eagles medium (SH30243.01, HyClone, Shanghai, China) containing 10% fetal bovine serum (FSS500, ExCell Bio, Suzhou, China) and 1% penicillin/streptomycin (Thermo Fisher Scientific, Billerica, MA, USA) at 37 °C and 5% CO_2_. Transfections were performed by electroporation using the Bio-Rad Gene Pluser X cell system (Bio-Rad, Hercules, CA, USA) with 4 mm cuvettes. A voltage step pulse (180 V, 25 ms) was used for transfecting HEK293 cells. All experiments were carried out 24 h post-transfection [41].

### 2.7. Calcium Imaging

Calcium imaging was performed with a Zeiss observer Z1 microscope equipped with an X-cite 120-Q light source (Lumen Dynamics, Waltham, MA, USA), a 40× oil objective (NA1.3), and a sCMOS digital camera (ORCA-Flash4.0 V3, Hamamatsu Photonics, Hamamatsu City, Japan). Cell-seeded coverslips were mounted into an open-topped chamber and loaded with Ca^2+^ imaging buffer, which contained 107 mM NaCl, 7.2 mM KCl, 1.2 mM MgCl_2_, 11.5 mM glucose, and 20 mM HEPES-NaOH (pH 7.2). Time-lapse imaging was controlled by SlideBook 6.0 software. Filters for TuNer (490–510 nm Ex and 520–550 nm Em for mNeonGreen; 426–450 nm Ex and 457–481 nm Em for mTurquoise) and mScarlet (554–590 nm Ex and 602–662 nm Em) were used [42]. Images were acquired every 2 s. The fluorescence intensity from the regions of interest was exported and further analyzed with MATLAB R2014a (Mathworks, Natick, MA, USA) and plotted with GraphPad Prism 7. The ER Ca^2+^ level was indicated by the ratio of F_mNeonGreen_ to F_mTurquoise_. At least three repeats were performed for each experiment. All traces are shown as the mean ± standard error of the mean (SEM).

### 2.8. Differential Expression Analysis

Differential gene expression analysis was performed using DESeq2 (version 1.28.1). The heatmaps were plotted using ComplexHeatmap (version 2.6.2) [43]. Hierarchical clustering is carried out only within the TE family with counts per million (CPM) greater than 2, while K-means clustering was used to divide them into four modules separated by horizontal lines.

### 2.9. Embryo Tyramide Signal Amplification Fluorescence In Situ Hybridization

Embryos were collected for tyramide signal amplification fluorescence in situ hybridization (TSA-FISH) based on the development stages and fixed with 600 μL heptane and 200 µL 4% formaldehyde [44]. The designed primers containing the flanking T7 promoter elements are listed in Table S2. FISH of *Drosophila* embryos was performed according to the method described by Jandura et al. [45]. Biotin-conjugated mouse monoclonal anti-digoxin (DIG) antibody (200-062-156, Jackson ImmunoResearch) was used at a dilution of 1:400, and streptavidin HRP solution (S991, Invitrogen, Shanghai, China) was used at a dilution of 1:1000 to detect the DIG-labeled probes. DAPI solution (1 μg/mL) was used to detect DNA. Antibodies were detected by cyanine 3-conjugated tyramide diluted 1:80 in an activation buffer containing 0.006% H_2_O_2_. Microscopy imaging was performed using the LSM 880 laser confocal fluorescence microscope (Zeiss, Shanghai, China). Image processing and fluorescence intensity analysis were performed using Fiji software (version 1.53c).

### 2.10. Statistics

All quantitative data are presented as mean ± S.D. of at least three independent biological repeats. Comparisons between two groups were conducted using unpaired Student’s *t*-test, and comparisons among multiple groups were analyzed with One-way ANOVA.

## 3. Results

### 3.1. Phenotypic Consequences in Aneuploidy

**Figure 1:**
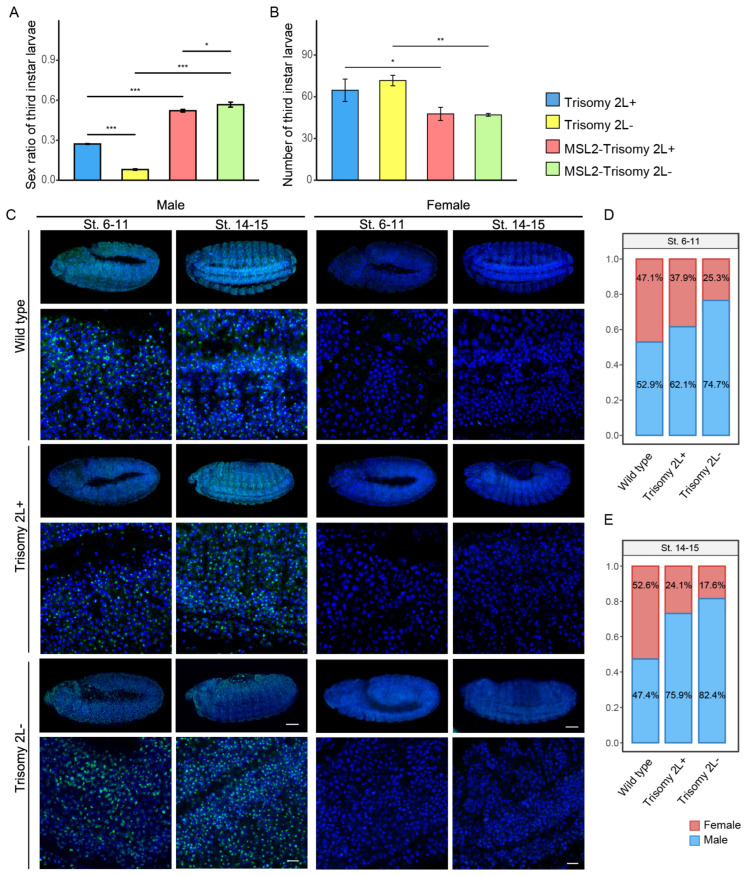
The sex ratios and the number of third instar larvae and embryos. (**A**,**B**) The sex ratios (**A**) and the number of trisomy 2L+, trisomy 2L-, MSL2-trisomy 2L+, and MSL2-trisomy 2L- larvae (**B**). The sex ratio is the number of females divided by the number of males multiplied by 100 percent. Data are expressed as the means of 3 independent experiments. Error bars indicate mean ± standard deviation (S.D.). The two-tailed Student’s *t*-test was used to analyze statistical variance * *p* < 0.05, ** *p* < 0.01, *** *p* < 0.001; non-significant comparisons are omitted. (**C**) Immunolocalization of H4K16Ac in the embryo. Wild type and trisomy 2L embryos were stained with an antibody and DAPI to visualize H4K16Ac (green) and DNA (blue), respectively. The sex and the developmental stage of the samples are shown above, and the genotypes of the samples are shown on the left. Scale bars, 50 and 10 μm. (**D**,**E**) Stacked percentage barplots of female (red) and male (blue) according to relative fluorescence intensity at Stages 6–11 (**D**) and 14–15 (**E**) of embryonic development in wild type and trisomy 2L files.

Aneuploidies were generated through reciprocal crosses, as outlined in Figure S1 (y; C(2L) dp; F(2R) bw females × Canton S males and Canton S females × y; C(2L) dp; F(2R) bw males). In contrast to diploidy, monosomy 2L exhibited morphological abnormalities during embryonic development and failed to develop into larvae, while trisomy 2L failed to mature into adults. In addition to the lethality of aneuploidy, we observed a significant decrease in the sex ratios (female to male) and the total number of third instar larvae in trisomy 2L (Figure 1A,B). The sex ratio was significantly lower in trisomy 2L- (0.080 ± 0.005) compared to trisomy 2L+ (0.272 ± 0.003), and both were significantly lower than that in diploidy (1.06 ± 0.03) (Figure 1A). Moreover, there was no significant difference in the number of third instar larvae between trisomy 2L+ (64.667 ± 8.083) and trisomy 2L- (71.667 ± 3.786) (Figure 1B). Subsequently, we ectopically expressed the MSL2 protein in trisomy 2L (Figures S2 and S3). The sex ratios of third instar larvae significantly increased in both MSL2-trisomy 2L+ (0.522 ± 0.010) and MSL2-trisomy 2L- (0.567 ± 0.019), although they did not recover to diploid levels (Figure 1A). In addition, the difference in the sex ratio between MSL2-trisomy 2L+ and MSL2-trisomy 2L- was also alleviated (Figure 1A). However, the total number of larvae was decreased compared to no ectopic MSL2 expression (Figure 1B).

A previous study showed that H4K16ac was more enriched in male embryos than in female embryos [38]. Therefore, the sex ratios of the embryos were analyzed based on varying levels of H4K16Ac from immunolocalization (Figure 1C and Figure S4). At Stages 6–11 and 14–15 of embryonic development, sex ratios in diploidy were consistently close to 0.5 (Figure 1D,E). In both instances of trisomy 2L, sex ratios decreased as the embryos developed, gradually approaching levels observed in the third instar larvae (Figure 1A,D,E). Sex ratios were lower in trisomy 2L- than in 2L+ during these embryonic developmental stages. These results indicate that the sex ratio in trisomy 2L decreased during embryonic development, suggesting a continuous change. Differences in sex ratios between trisomy 2L- and 2L+ persisted across the embryonic developmental stages.

The impact of the MSL complex on global gene expression and the potential target genes of MSL2 have been identified in aneuploidy [36]. To further determine its contribution to the effects of parental genomic imbalance, expression profiles were examined when MSL2 was overexpressed in aneuploid flies using TSA-FISH in reciprocal trisomy 2L and MSL2-trisomy 2L.

**Figure 2:**
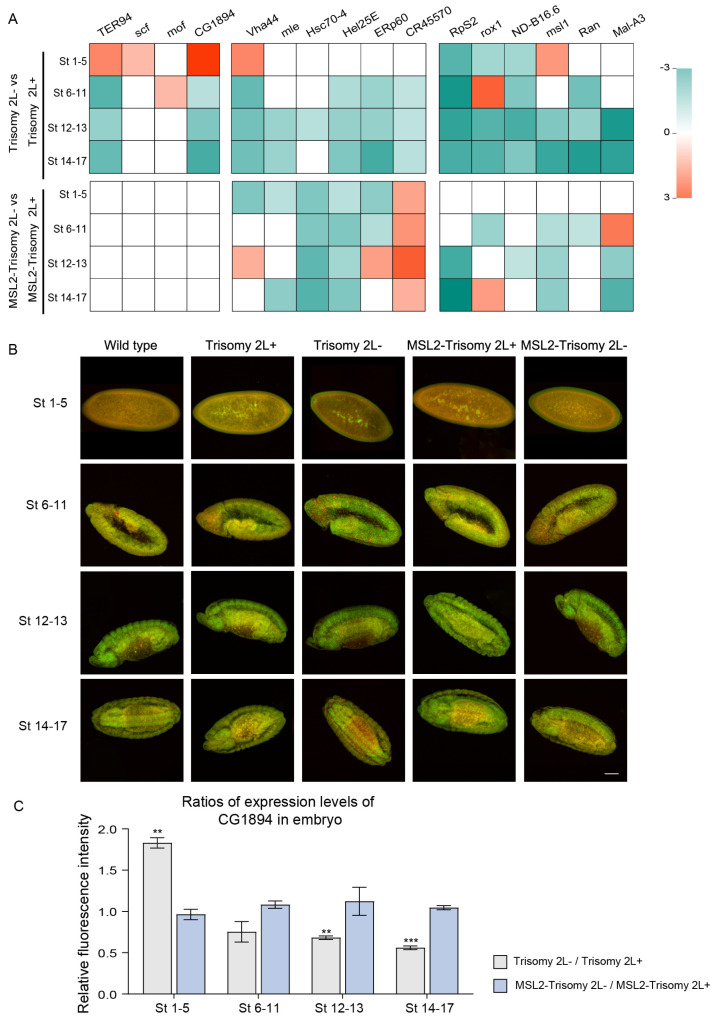
Expression patterns of candidate genes in the embryo. (**A**) Heatmap of log10 (*p* value). When the expression levels of genes in trisomy 2L- were significantly upregulated compared with trisomy 2L+, −log10 (*p*-value) was calculated. Conversely, when the expression levels of genes in trisomy 2L- were significantly downregulated compared with trisomy 2L+, log10 (*p* value) was calculated. This evaluation was the same as for the comparison of MSL2-trisomy. The log10 (*p* value) of nonsignificant DEGs was set to 0 and rendered white in the heatmap. Genes are shown above, and the genotype and the stages of the samples are shown on the left. (**B**) Subembryonic distribution of *CG1894* in wild type, trisomy 2L, and MSL2-trisomy 2L. The genotype of the sample is shown above, and the stage of embryonic development is shown on the left. Green is the DNA signal (DAPI), and red is the RNA probe signal (cy3). Scale bar, 30 μm. (**C**) The ratios of *CG1894* expression levels of trisomy 2L- to trisomy 2L+ and MSL2-trisomy 2L- to MSL2-trisomy 2L+. The relative fluorescence intensity was calculated as the ratio of the RNA signal to the DAPI signal. Error bars indicate mean ± S.D. The one-sample Wilcoxon test was used to analyze statistical variance and the hypothesis value was set to 1. ** *p* < 0.01; *** *p* < 0.001; non-significant comparisons are omitted.

**Figure 3:**
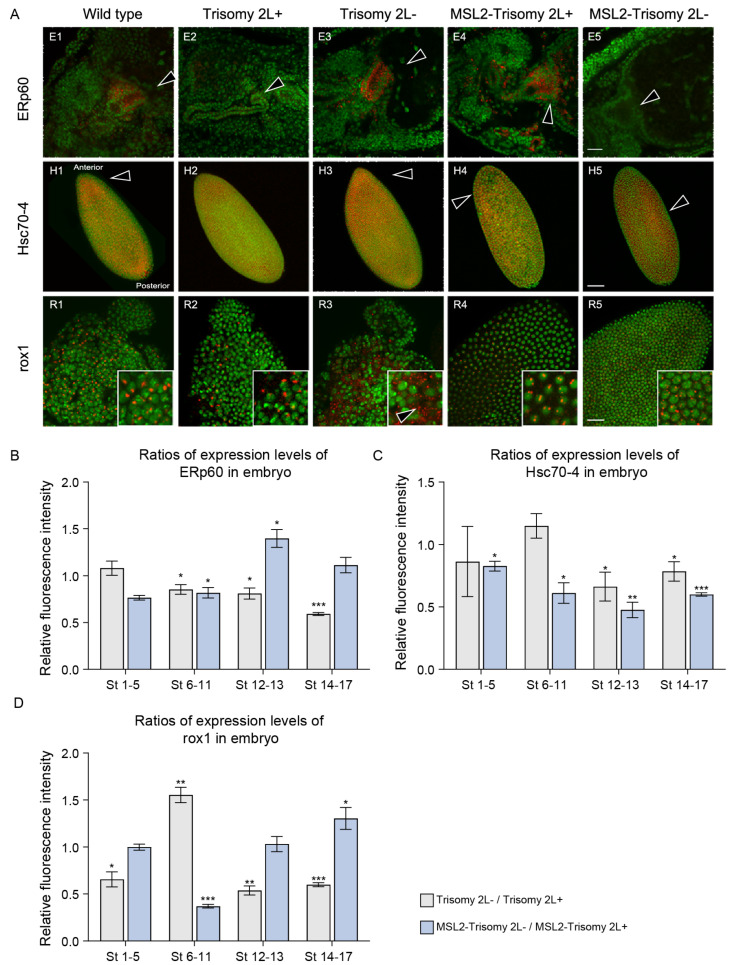
Transcript localization and relative fluorescence intensity of *ERp60*, *Hsc70-4*, and *roX1*. (**A**) Changes in transcript localization of *ERp60*, *Hsc70-4*, and *roX1* RNA in wild type, trisomy 2L+, trisomy 2L-, MSL2-trisomy 2L+, and MSL2-trisomy 2L-. The genotype of the sample is shown above, and the type of RNA probe is shown on the left. Green is the DNA signal, and red is the RNA probe signal. The arrowheads in E1–E5 present the location of the foregut. E1–E5, Stages 14–17; H1–H5, Stage 5; R1–R5, Stages 6–11. Scale bars, ERp60 and rox1, 30 μm; Hsc70-4, 80 μm. (**B**–**D**) The ratios of *ERp60* (**B**), *Hsc70-4* (**C**), and *rox1* (**D**) expression levels of trisomy 2L- to trisomy 2L+ and MSL2-trisomy 2L- to MSL2-trisomy 2L+. The relative fluorescence intensity was calculated as the ratio of the RNA signal to the DAPI signal. Error bars indicate mean ± S.D. The one-sample Wilcoxon test was used to analyze statistical variance and the hypothesis value was set to 1. * *p* < 0.05; ** *p* < 0.01; *** *p* < 0.001; non-significant results are omitted.

All 16 detected genes showed differential expression levels across embryonic developmental stages between trisomy 2L+ and 2L-, determined by calculating the ratios of trisomy 2L- to 2L+. Compared to trisomy 2L+, trisomy 2L- exhibited a significant upregulation of *Vha44*, *TER94*, *CG1894*, *msl-1*, and s*cf*, and a downregulation of *RpS2*, *roX1*, and *ND-B16.6* at Stages 1–5 (Figure 2 and Figures S5–S7). In subsequent embryonic development stages, the majority of these genes (seven out of eight) were also downregulated in trisomy 2L-, indicating more pronounced changes in trisomy 2L- compared to trisomy 2L+ (Figure 2A and Figures S5–S7). These factors have been reported to play important roles in gene expression, protein translation, and other biological processes [46,47,48]. Non-differentially expressed genes (non-DEGs) in Stages 1–5 showed significant changes in Stages 6–17 (8 out of 16 genes). The expression levels of *Hel25E*, *ERp60*, *CR45570*, and *Ran* showed significant downregulation during Stages 6–17, whereas *mof*, *Hsc70-4*, and *Mal-A3* exhibited significant changes only at Stages 6–11, 12–13, and 12–17, respectively (Figure 2A and Figure 3 and Figure S5–S7). *roX1* exhibited differential expression levels at all stages between trisomy 2L+ and 2L-, with inhibition observed in trisomy 2L- at most stages (Figure 2A and Figure 3).

The ectopic expression of MSL2 did not result in significant differences in the expression levels of *TER94*, *scf*, *mof*, and *CG1894* (4 out of 16 genes) across embryonic development stages (Figure 2 and Figure S5). The expression levels of *Vha44*, *mle*, *Hsc70-4*, *Hel25E*, *ERp60*, and *CR45570* in MSL2-trisomy 2L- were closer to the diploidy than trisomy 2L+ (Figure 2A and Figures S6 and S14). However, the difference in the expression levels of *mle*, *Hsc70-4*, *Hel25E*, *ERp60*, and *CR45570* between reciprocal crosses was evident from Stages 1–5 and co-persisted through subsequent developmental stages, except for *Vha44* (Figure 2A and Figure S6). *RpS2*, *roX1*, *ND-B16.6*, *msl-1*, *Ran*, and *Mal-A3* showed no differences in MSL2-trisomy 2L at Stages 1–5 (Figure 3 and Figure S7).

In summary, the expression levels of sixteen genes were assessed across four main developmental stages, totaling 64 loci. Trisomy 2L- exhibited 46 significantly different loci (about 71.9%) compared to trisomy 2L+, with 39 of them being downregulated (Figure 2A). These results identified distinct gene expression patterns between trisomy 2L+ and 2L-, with trisomy 2L- showing a greater number of significantly differentially expressed genes (DEGs), further emphasizing the genetic divergence between the two conditions. Additionally, there were 31 (about 48.4%) significantly different loci between MSL2-trisomy 2L+ and 2L-, with 23 of them being downregulated (Figure 2A). Following MSL2 overexpression, 15 loci that were not significantly differentially expressed indicated a reduction in reciprocal effects by 32.6% (Figure 2A).

More interestingly, the subcellular locations of *ERp60*, *Hsc70-4*, and *roX1* exhibited differences between trisomy 2L+ and trisomy 2L- (Figure 3A). At Stages 14–17, *ERp60* was enriched in the foregut in diploidy and trisomy 2L- but not in trisomy 2L+ (Figure 3A). *Hsc70-4* exhibited apical and basal enrichment in diploidy and trisomy 2L- whereas it appeared evenly distributed in trisomy 2L+ at Stage 5 (Figure 3A). In brain neuroblasts, *roX1* in trisomy 2L+ was located in the perinuclear region, whereas it showed intranuclear accumulation in diploidy and trisomy 2L- at Stages 6–11 (Figure 3A). These results demonstrate that reciprocal crosses could induce expression levels or specific subcellular or embryonic localization patterns of genes, suggesting potential correlations with regulatory mechanisms.

Similarly, differences were observed in the subcellular localization of *ERp60* and *Hsc70-4* between MSL2-trisomy 2L+ and 2L-. At Stages 14–17, *ERp60* transcripts showed significant enrichment in the foregut of diploidy and MSL2-trisomy 2L+ but not in MSL2-trisomy 2L- (Figure 3A). *Hsc70-4* transcripts exhibited local enrichment in all instances of MSL2-trisomy 2L at Stage 5 (Figure 3A). Thus, reciprocal effects in aneuploidy seemed to diminish following MSL2 expression, as evidenced by decreased differences in expression levels and transcript localization. In addition, dysregulation of the expression levels of *RpS2*, *msl-1*, and *Mal-A3* and the abnormal localization of *ERp60* in MSL2-trisomy 2L- may be the key factors causing the growth and developmental disorders.

In general, reciprocal effects were evident in both the expression levels and the locations of key genes in aneuploidy. The expression levels of 16 detected genes in trisomy 2L- were mostly lower than those in trisomy 2L, which partly explains the more pronounced downregulation observed in the sex ratios of trisomy 2L- (Figures S13–S15). The differential expression and localization of genes during early embryonic development also illustrated the potential influence of maternal effects on reciprocal effects in aneuploidy. The ectopic expression of MSL2 protein in trisomy 2L led to an increase in sex ratios for both genotypes, suggesting a role for MSL2 in modulating the survival number under aneuploid conditions. The number of DEGs between MSL2-2L+ and MSL2-2L- was also reduced, and the effect of MSL2 on sex ratio may be related to changes in the expression levels and localization of these genes.

### 3.2. Maternal Genes Influenced by MSL2 in Unbalanced Genomes

**Figure 4:**
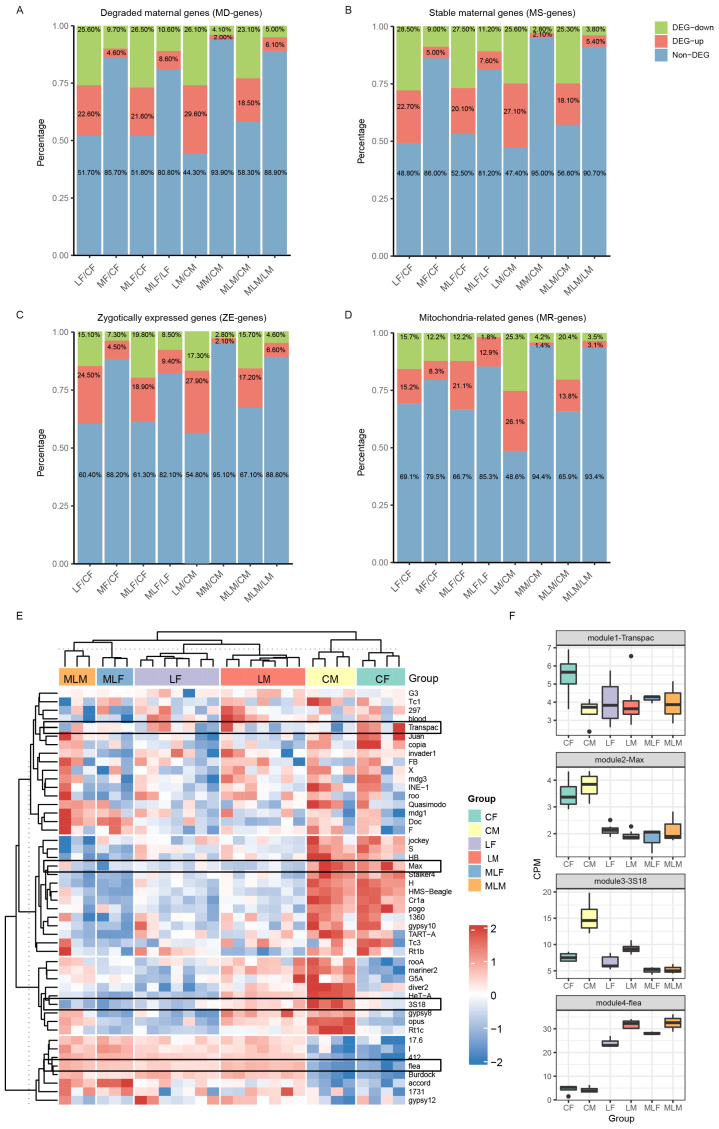
The expression levels of genes and TEs in aneuploidy. (**A**–**D**) The proportion of three modes of gene alterations: upregulated genes (red), downregulated (green), and non-significantly differential genes (blue). The numbers of the three gene types are 2637, 2118, and 820, respectively and they include (**A**) degraded maternal genes (MD-genes), (**B**) stable maternal genes (MS-genes), (**C**) zygotically expressed genes (ZE-genes), and (**D**) mitochondria-related genes (MR-genes). The samples are shown, including trisomy 2L and wild type (LF vs. CF), over-expressed MSL2 and wild type (MF vs. CF), MSL2-trisomy 2L and wild type (MLF vs. CF), MSL2-trisomy 2L and trisomy 2L (MLF vs. LF), trisomy 2L and wild type (LM vs. CM), over-expressed MSL2 and wild type (MLM vs. CM), MSL2-trisomy 2L and wild type (MLM vs. CM), and MSL2-trisomy 2L and trisomy 2L (MLM vs. LM). The percentages are shown on the left. (**E**,**F**) Differential expression of (TE) families of aneuploidy compared to diploidy. (**E**) Heatmap showing the expression levels of TEs in the groups. CF, wild type female; CM, wild type male; LF, trisomy 2L female; LM, trisomy 2L male; MLF, MSL2-trisomy 2L female; and MLM, MSL2-trisomy 2L male. Each column is a biological replicate and each row is a TE family. Hierarchical clustering is carried out only within the TE family with CPM greater than 2. K-means clustering is used to divide selected TE families into four modules separated by horizontal lines. (**F**) The expression of the selected TE families from four modules. The middle lines in the boxplots indicate the medians. Whiskers are the line segments from the box boundary to the maximum and minimum non-outliers. Black dots indicate outliers that lie outside of the whisker.

During the study of maternal genes in *Drosophila*, researchers identified two distinct classes of maternal genes, degraded maternal genes (MD-genes) and stable maternal genes (MS-genes). In addition, zygotically expressed genes (ZE-genes) were also screened [49]. MD-genes undergo degradation during the MZT, whereas MS-genes maintain stable expression profiles throughout embryonic development. To investigate how genomic imbalance and MSL2 overexpression affect maternal gene expression patterns, we conducted an integrative analysis of our transcriptome and maternal gene data and compared the differential expression of the three gene categories between aneuploidy and diploidy (Figure 4A–C). The DEGs included upregulated DEGs (DEGs-up) and downregulated DEGs (DEGs-down). We primarily analyzed the transcriptome data of diploidy, trisomy 2L, MSL2 overexpression, and MSL2-trisomy 2L, enabling a comprehensive evaluation of how MSL2 impacts maternal genes under both diploid and aneuploid conditions (Figure 4A–C).

We found that the number of DEGs in aneuploid males (LM vs. CM) was higher than that in females (LF vs. CF). In males, the proportion of DEGs of both MD- and MS-genes was up to 50%, exceeding the proportions observed in females (Figure 4A,B). Specifically, the differential expression of MS-genes was more pronounced in males, accounting for 55.7%, compared to 48.3% in females. Following MSL2 overexpression in trisomy 2L females (MLF vs. LF), the proportion of DEGs in MD-genes did not significantly change compared to trisomy 2L and there was a reduction in the DEGs of MS- and ZE-genes (Figure 4A–C). Conversely, in males (MLM vs. LM), there was a significant reduction in differential expression across all three gene types, with non-DEGs increasing by approximately 10% for the two types of maternal genes. These results clearly suggest that MSL2 can reduce the number of DEGs in aneuploidy. In MSL2-trisomy males, maternal gene expression levels more closely approximated normal levels compared to females, possibly accounting for the higher proportion of males among larvae. Additionally, the proportion of DEGs-up varied between males and females, aligning with previous research [36].

In diploid males, where approximately 93.9% of MD-genes and 95% of MS-genes were unaffected, the effects of overexpressing MSL2 on the three types of genes were less pronounced compared to females (MM vs. CM and MF vs. CF), likely due to the presence of endogenous MSL2 in males (Figure 4A–C). The proportions of DEGs in trisomy were slightly higher compared to the smaller changes observed in diploidy (MLM vs. LM). These data illustrate that the effects in trisomy 2L, including gene dosage and inverse dosage effects, influenced genome-wide gene expression. In addition, it was further confirmed that MSL2 interacts with the inverse dosage effect and plays a role in this regulatory mechanism.

### 3.3. Mitochondria-Related Genes Influenced by MSL2 in Unbalanced Genomes

In most organisms, such as *Drosophila* [50], mitochondria are inherited through the maternal line. In this study, the mitochondria-related genes (MR-genes) were also analyzed to further determine their possible roles in unbalanced genomes (Figure 4D). Changes in MR-genes exhibited sex-specific differences, akin to the three types of genes analyzed. A greater proportion of MR-genes was differentially expressed in males (51.4%, 2LM vs. CM) than in females (30.9%, 2LF vs. CF). Furthermore, the proportions of up- and down-regulated MR-genes were nearly identical across sexes, with 26.1% and 25.3% in males, and 15.2% and 15.7% in females, respectively.

Following MSL2 overexpression, the reduction in differentially expressed MR-genes among MSL2-trisomy 2L females compared to wild type females was less than 3% (MLF vs. CF). In MSL2-trisomy 2L females, the DEGs-up increased significantly (21.1%), and more than DEGs-down (12.2%), which were basically equal in trisomy 2L (LF vs. CF). Conversely, the proportion of non-DEGs in males notably increased from 48.6% in trisomy 2L (LM vs. CM) to 65.9% in MSL2-trisomy 2L (MM vs. CM), suggesting a possible regulatory role of MR-genes in the sex ratio of larvae during reciprocal crosses. Notably, there was a significant decrease in both DEGs-up and DEGs-down in MSL2-trisomy 2L males, particularly a marked reduction from 26.1% to 13.8% in DEGs-up.

When MSL2 was overexpressed in diploidy compared with the wild type (MF vs. CF and MM vs. CM), more MR-genes were dysregulated in females (MF vs. CF, 20.5%) than in males (MM vs. CM, 6.6%). This difference is attributed to the presence of the endogenous MSL complex in males, which also demonstrates that the complex has some buffering effects on gene expression. When MSL2 was ectopically expressed in aneuploidy, it was found that the upregulated genes increased and the downregulated genes decreased in females and males compared with the above MSL2 diploid conditions. However, the total number of DEGs was relatively stable in males but not in females. When MSL2 was ectopically expressed in aneuploidy, the number of DEGs-up increased and the DEGs-down decreased in females and males compared with the above MSL2 diploid conditions. The DEGs-up increased and that of DEGs-down decreased in females (MLF vs. LF), while the total number of DEGs was relatively stable. All of these results suggest that MSL2 elicits a much greater influence on gene expression in females than in males, including the expression of maternal genes and MR-genes.

During MZT, a portion of the oocyte-loaded maternal products are removed and the zygotic genome is activated. In *Drosophila*, early embryogenesis is characterized by 14 rapidly synchronized nuclear divisions, and the zygotic genome is nearly quiescent until the seventh or eighth nuclear division. Based on the characteristics in *Drosophila* embryogenesis, the dynamic expression levels of maternal genes were analyzed by collecting embryos at 0.75 h (nearly pre-MZT and pre-ZGA), 3 h (post-ZGA), and 6 h (post-MZT) postfertilization. To further verify the expression levels of maternal genes in reciprocal crosses in addition to the above RNA-seq results, representative maternal and MR-genes were selected to verify the differences in expression between trisomy 2L and MSL2-trisomy 2L by using RT-PCR, including *Jafrac1*, *mtrm*, *pgc*, *26-29-p*, *Tub67C*, *ArgRS,* and *osk* (Figure S8). We first validated the expression levels of MD-genes in the three stages in the wild type, demonstrating high expression levels pre-MZT and a near absence of expression after ZGA. The above results aligned with the definition of MD genes in the database (no. GSE143821), which states that MD genes are degraded during the MZT. To observe the differences between reciprocal crosses more intuitively, the ratios of the relative expression levels between trisomy 2L+ and 2L- (Figure S8A–C), as well as between MSL2-trisomy 2L+ and 2L- (Figure S8B–D), were determined. Consistent with the expected results, six tested genes were differentially expressed between trisomy 2L+ and 2L- before ZGA, decreasing to two after MSL2 overexpression. These results not only show the different changes in maternal and mitochondrial genes between trisomy 2L+ and 2L- but also prove that MSL2 has a positive effect on maternal expression in embryos. After ZGA, the expression levels of only four genes were similar between MSL2 trisomy 2L+ and 2L-. After MZT, only one gene, *pgc,* was detected. The above results indicate that MSL2 had a greater impact on maternal genes in the early stages of embryonic development, while the impact was gradually weakened after ZGA.

We investigated the inheritance and expression dynamics of MR-genes in *Drosophila*, focusing on their roles in sex-specific gene regulation and embryonic development. We found that MR-genes are predominantly maternally inherited and exhibit sex-specific differences in expression, with a higher proportion of DEGs in males compared to females. The overexpression of MSL2 led to a significant increase in upregulated MR-genes and a decrease in downregulated MR-genes, particularly in females. This suggests that MSL2 plays a more substantial role in female gene expression, influencing both maternal and MR-genes. Our analysis of gene expression during early embryogenesis revealed that MSL2 has a pronounced impact on maternal gene expression before ZGA, which diminishes post-ZGA. Collectively, these findings underscore the potential impact of MSL2 on maternally expressed genes.

### 3.4. Transposable Elements Influenced by MSL2 in Unbalanced Genomes

Differential expression analysis was performed for TE families, clustering them into four distinct modules (Figure 4E). The TE families in the first and second modules exhibited downregulation in trisomy 2L and MSL2-trisomy 2L compared to the wild type. In the third module, the expression levels of these TE families differed between male and female in the wild type but remained downregulated in all aneuploidies. Indeed, the downregulation of TEs in these three modules was consistent with the general expression patterns observed in trisomy, potentially influenced by inverse dosage effects. The TE families in the fourth module were upregulated in contrast to the overall gene expression patterns. Subsequently, we selected representative TE families from these four modules and detected their expression pattern across embryonic development stages using TSA-FISH to verify our analyses and further confirm their potential functions.

**Figure 5:**
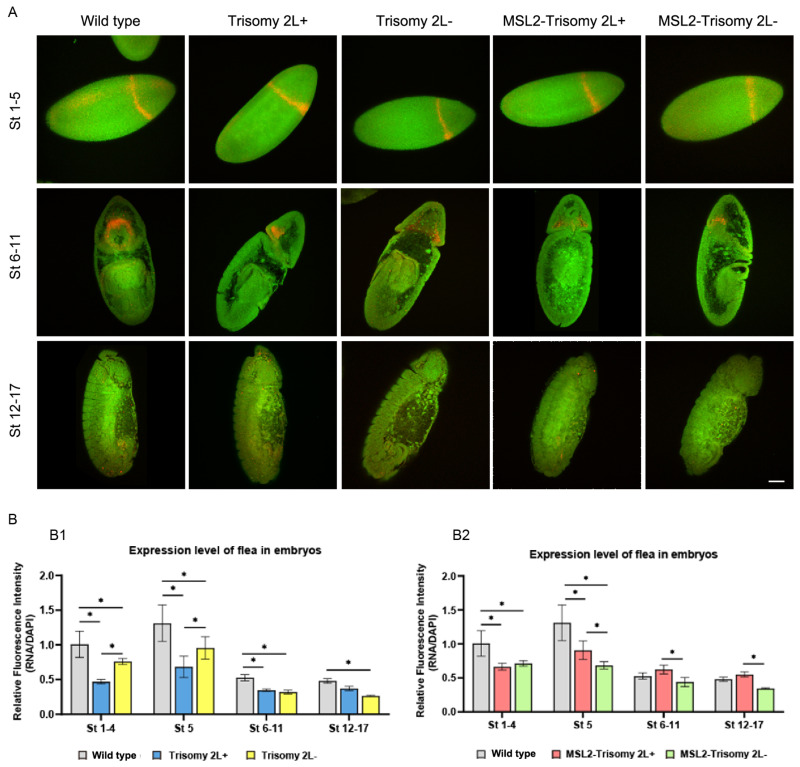
Embryo FISH of the TE *flea*. (**A**) The expression patterns of the TE *flea* in the entire embryo. The genotype of the sample is shown above, and the development stages of the sample are shown on the left. Green is the DNA signal (DAPI), and red is the RNA probe signal (cy3). Scale bar, 63 μm. (**B**) Relative fluorescence intensity of the *flea* signal in trisomy 2L and MSL2-trisomy 2L compared with normal diploidy. The relative fluorescence intensity was calculated as the ratio of the RNA signal to the DAPI signal. (**B1**) Relative fluorescence intensity of *flea* signal in wild type and trisomy 2L contained by reciprocal crosses. (**B2**) Relative fluorescence intensity of the *flea* signal in wild type and MSL2-trisomy 2L contained by reciprocal crosses. The two-tailed Student’s *t*-test was used to analyze statistical variance. * *p* < 0.05; non-significant comparisons are omitted.

In the fourth module, *flea* localization was significantly enriched during embryonic development (Figure 5A). It exhibited apical enrichment and anterior localization at Stages 1–5 in the wild type. During the formation of the cephalic furrow in the appearance of the stomodeum, the *flea* was enriched in the procephalon near the cephalic furrow (Figure 5A). However, its localization did not change in aneuploidy. At Stages 1–5, the expression levels varied between trisomy 2L+ and 2L-, with the difference disappearing in subsequent developmental stages. The expression levels of trisomy 2L were lower than those in the wild type (Figure 5B1). No differences in expression levels were observed between MSL2-trisomy 2L+ and 2L- at Stages 1–4, with slight differences appearing in subsequent developmental stages, and their expression levels gradually approached normal levels (Figure 5B2).

In the first module, *Transpac* was downregulated during embryonic development in trisomy 2L+ and MSL2-trisomy 2L+ in third instar larvae, consistent with the RNA-seq results. Notably, *Transpac* expression levels were significantly downregulated in trisomy 2L+ and upregulated in trisomy 2L- at Stages 1–5 (Figure S9B1). Following MSL2 over-expression, there was no difference between trisomy 2L+ and 2L- at all stages, but the expression levels did not return to normal (Figure S9B2). In the second module, *3S18* has been reported to be involved in the development of eyes in *Drosophila* [51]. At Stages 1–4, expression levels of *3S18* varied in reciprocal crosses, depending on MSL overexpression (Figure S10B). MSL2 overexpression resulted in the expression levels of *3S18* in trisomy closer to those of wild type, especially in MSL2-trisomy 2L- (Figure S10B1). Moreover, differential expression was observed between trisomy 2L+ and 2L- across embryonic development stages. Differences between MSL2-trisomy 2L+ and MSL2-trisomy 2L- disappeared at Stage 5, whereas they persisted until Stages 6–11 between trisomy 2L+ and 2L- (Figure S10B). A smaller difference was observed in the expression levels of *Max* in the third module in trisomy 2L than MSL2-trisomy 2L, with trisomy 2L- approaching normal levels at Stages 1–5 (Figure S11B1). Although trisomy 2L- deviated from the normal levels in subsequent developmental stages, the difference between trisomy 2L+ and 2L- disappeared. This difference persisted across all embryonic developmental stages between MSL2-trisomy 2L+ and 2L-, although the expression levels gradually approached those of the wild type (Figure S11B2).

In summary, the reciprocal effects in TEs changed following MSL2 overexpression. The expression levels of all transposons were different between trisomy 2L+ and trisomy 2L-. Differences in *Transpac* expression levels between MSL2-trisomy 2L+ and 2L- at all stages, as well as differences in *3S18* and *flea* expression levels at Stage 5, disappeared. The expression levels of some TEs in MSL2-trisomy 2L were closer to those of the wild type. These results indicate the correlation between TE and reciprocal effects in aneuploidy, highlighting the beneficial role of MSL2. Moreover, the changes in these TEs were related to some functions that require further investigation.

### 3.5. Regulation of Endoplasmic Reticulum Calcium Homeostasis by MSL2 Targets in Aneuploidy

*ERp60* was screened from a set of DEGs identified between aneuploid and diploid genomes [36]. Consistently, we discovered that the expression levels and location of *ERp60* were significantly altered in aneuploid embryos. Furthermore, the ER serves as a crucial intracellular Ca^2+^ reservoir, with *ERp60* acting as an ER resident disulfide isomerase in *Drosophila* [48].

**Figure 6:**
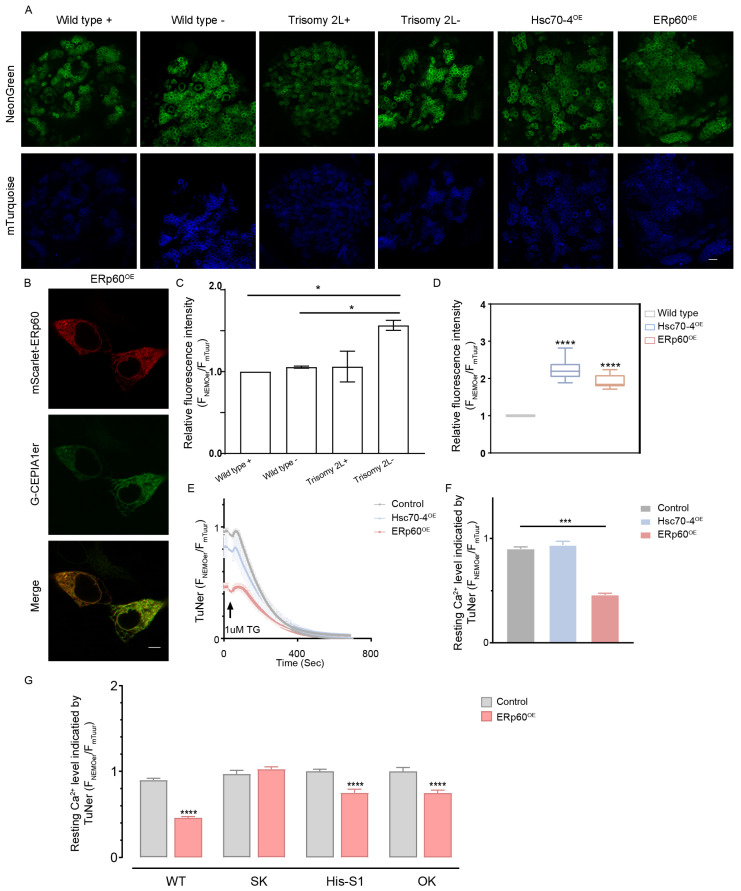
ER Ca^2+^ levels measurements by mTurquoise2- NEMOer (TuNer) in HEK293 cells and *Drosophila*. (**A**) Ca^2+^ levels in the larvae brains of wild type, trisomy 2L, obtained with reciprocal crosses, Hsc70-4^OE^, and ERp60^OE^ measured using TuNer indicators. The horizontal axis shows the genotypes of the samples, which are wild type and trisomy 2L, respectively, and the left vertical axis shows different fluorescence types. Wild type+ and Wild type- denote wild type as paternal and maternal, respectively. The green is the fluorescence signal of NeonGreen, and the blue is the fluorescence signal of mTurquoise. Scale bar, 10 μm. (**B**) Cellular co-localization of *ERp60* with ER in HEK293 cells. mScarlet, the red fluorescent protein, revealing the location of *ERp60*; G-CEPIA1er, the green fluorescent protein for imaging the location of ER. Scale bar, 5 μm. (**C**) Statistics of ER Ca^2+^ levels in larvae brains of wild type and trisomy 2L. The Ca^2+^ levels are indicated by the ratio of F_mNeonGreen_/F_mTurquoise_. (**D**) Ca^2+^ levels in the larvae brains of control (wild type), Hsc70-4^OE,^ and ERp60^OE^ were measured using TuNer. (**E**) Representative traces. Cells stably expressing TuNer, the latest ER Ca^2+^ indicator, were correspondingly transfected with blank control (HEK293 cells, gray), ERP60^OE^ (red), or HSC70-4^OE^ (blue). After recording of basal Ca^2+^ level, thapsigargin (TG, 1 μM), a blocker of SERCA, was then added to deplete the ER Ca^2+^ store. (**F**,**G**) Statistics of ER Ca^2+^ resting level. SK, STIM knock out; OK, Orai knock out; and HIS-STIM1, cells with STIM1 overexpression (n = 3, at least 15 cells in each repeat). Error bars denote mean ± S.D. The two-tailed Student’s *t*-test was used to analyze statistical variance. * *p* < 0.05; *** *p* < 0.001; **** *p* < 0.0001; non-significant comparisons are omitted.

To further identify its related regulatory functions in unbalanced genomes, we investigated Ca^2+^ levels in aneuploidy using the most advanced ER-targeted Ca^2+^ indicator (Figure 6A). Researchers developed a highly dynamic and sensitive green genetically encoded calcium indicator (GECI) known as mNeonGreen-based Ca^2+^ indicator for ER (NEMOer), and its ratiometric version mTurquoise2-NEMOer (TuNer) was used in this study [52]. We transgenically expressed TuNer in *Drosophila* using the GAL4/UAS system to drive its expression in the nervous system. It was found that the Ca^2+^ levels of trisomy 2L- declined compared to those of the wild type (Figure 6A–C), illustrating the changes in ER Ca^2+^ homeostasis in imbalanced genomes.

To explore gene function, plasmids with the red fluorescent protein mScarlet expressing *ERp60* and *Hsc70-4* were constructed (Figure S12A,B). After testing their localization in HEK293 cells, transgenic *Drosophila* overexpressing *ERp60* and *Hsc70-4* were generated (Figure 6B). After elav-GAL4 *Drosophila* was used to activate the overexpression of *ERp60*, red fluorescence signals were detected in the brain (Figure S12C). Similarly, aneuploidies overexpressing *ERp60* and *Hsc70-4* were constructed through reciprocal crosses. Subsequently, we observed that both ERp60-trisomy 2L- and Hsc70-4-trisomy 2L- exhibited upregulated sex ratios compared to trisomy 2L and approached those of the wild type (Figure S12D,E). Importantly, sex ratios following ERp60 and Hsc70-4 overexpression exceeded those observed with MSL2 overexpression, implying a more direct influence of these genes on reciprocal effects.

It has been reported that *ERp57*, the human ortholog of *ERp60*, interacts with STIM1, inhibits the movement of STIM1 to puncta [53], and modulates sarco/ER Ca^2+^ ATPase (SERCA) function [54]. The dysfunction of STIM1 and SERCA may lead to ER Ca^2+^ dyshomeostasis, which is closely related to many diseases, such as neurodegenerative disorders and cancer [55,56]. To assess the role of *ERp60*, we measured ER resting Ca^2+^ levels in HEK293 cells overexpressing ERp60 (Figure 6A). Compared to cells expressing the blank control and Hsc70-4, those overexpressing ERP60 exhibited significantly reduced ER Ca^2+^ resting levels (Figure 6F). In the larval brain, overexpression of Hsc70-4 and ERp60 increased the ER Ca^2+^ resting levels (Figure 6E,F). The inconsistency of the results in cells may stem from the sustained high expression of these genes in *Drosophila*, leading to ER stress. These results showed the role of *Hsc70-4* and *ERp60* in regulating ER Ca^2+^ homeostasis, which might be correlated with its positive effect on the sex ratio of ERp60-trisomy 2L.

To further explore the mechanism of ER Ca^2+^ pool reduction, we examined the impact of ERp60 on SOCE. SOCE is crucial for maintaining ER Ca^2+^ homeostasis and is mediated by the STIM1 and Orai proteins, situated on the ER and plasma membrane, respectively [54]. STIM protein can sense the decrease in ER Ca^2+^ level, and then activates Orai and triggers the influx of extracellular Ca^2+^ ions. To investigate whether ERp60-mediated regulation of ER Ca2^+^ levels was implicated in these essential components of SOCE, ERp60 was overexpressed in cells, including STIM knock out (SK), Orai knock out (OK), and cells with STIM1 overexpression (HIS-STIM1), and resting Ca^2+^ levels were detected (Figure 6G). While *ERp60* significantly reduced Ca^2+^ levels in the wild type (WT), OK, and HIS-STIM1 cells, no reduction was observed in SK cells, suggesting that the regulation of *ERp60 of* the ER Ca^2+^ pool may depend on the STIM1.

All these results demonstrated that *ERp60* plays a crucial role in maintaining ERCa^2+^ homeostasis. It modulates ERCa^2+^ levels in both cellular and fly models through its interaction with STIM. This interaction might account for the observed increase in aneuploid survival following *ERp60* overexpression.

## 4. Discussion

Aneuploidy in *Drosophila* resulted in lethality and developmental issues during late embryonic and pupal stages, with significant gene expression changes. Reciprocal crosses produced aneuploidies with distinct sex ratios and gene expression patterns between trisomy 2L+ and trisomy 2L-, leading to decreased survival and female-to-male ratios in larvae. Importantly, MSL2 overexpression increased female survival and normalized sex ratios across crosses. TSA-FISH analysis revealed that trisomy 2L- embryos had more gene expression deviations from diploidy, and MSL2 overexpression reduced differential gene expression and improved cellular abnormalities. It is notable that most genes mentioned above altered in transcription levels and subcellular localization in early embryonic development stages, suggesting that this effect may be related to the influence of maternal expression. Aneuploidy and MSL2 overexpression led to complex regulation of maternal (MD-genes, MS-genes), MR-genes, and zygotic (ZE-genes) genes, with sex differences in gene expression potentially explaining higher male survival.

In a previous study, it was found that TEs are involved in genomic imbalance and aneuploidy development, with sensitivity to an inverse dosage effect [57]. In *Drosophila*, P elements trigger P-M hybrid dysgenesis. Maternally deposited P-element-induced wimpy testis (PIWI)-interacting RNAs (piRNAs) bond with PIWI proteins, directing them to silence the expression of TEs [58]. The piRNA-PIWI complex is crucial for epigenetic regulation, interacting with HP1a to enhance histone methylation at H3K9me [59]. Furthermore, PIWI also collaborates with the heat-shock organizing protein HOP to suppress phenotypic variation, with Hsc70 identified as an interactor [60,61,62]. It is intriguing to consider the potential underlying mechanisms by which Hsc70 may exert effects on these processes, and future research may elucidate the precise nature. The global expression of MSL2-trisomy was investigated by analyzing its transcriptome and found that TEs were not only affected by unbalanced genomes but also regulated by MSL2. This regulatory role may stem from its membership in macromolecular complexes or its involvement in multiple interactions. Similar to our previous study, the expression patterns of TEs were mainly downregulated in all aneuploidies, such as the second module, potentially related to the regulation via the inverse dosage effect. Interestingly, the TE families in the fourth module were all retrotransposons and exhibited upregulation in aneuploidy, particularly in MSL2-trisomy 2L. Given the inhibitory effect of transposition on gene expression, the upregulated expression of transposons in this module may be related to the downregulation of gene expression in aneuploidy, but further study is needed.

Interestingly, the overexpression of *ERp60* significantly increased the sex ratios of offspring in aneuploidy, with a more pronounced increase compared to MSL2 overexpression. This result suggested that *ERp60* might be a more direct factor modulated by MSL2 in influencing the viability and gene expression patterns of aneuploidy. Given that ER serves as the largest reservoir of calcium ions, we posited that the impact on the viability of ERp60-trisomy 2L may be linked to the regulation of Ca^2+^ homeostasis, supported by increased Ca^2+^ levels in aneuploid brains. Similarly, an elevation in intracellular cytoplasmic Ca^2+^ in cortical astrocytes was found in animal models of Down syndrome (DS) [63]. Ca^2+^ plays an important regulatory role in many biological processes, such as fertilization, growth, development, aging, and cell death [54,64,65]. We found that *ERp60* can regulate the ER Ca^2+^ pool by affecting SOCE, which might be dependent on STIM1. These research findings explained the role of *ERp60* in Ca^2+^ homeostasis and offered new insight into the interplay between Ca^2+^ homeostasis and genomic imbalance. The MSL complex is involved in achieving dosage compensation in unbalanced genomes, accompanied by epigenetic regulation mechanisms such as histone modification. Whether there are epigenetic factors involved in the regulation mediated by *ERp60*, such as microRNA, lncRNA, TEs, or RNA methylation, is a direction that will be interrogated in the future. In this study, we were unable to directly demonstrate that the effect of *ERp60* on Ca^2+^ homeostasis was responsible for the reduced survival number of aneuploidies. Thus, blue light-gated Ca^2+^ channels (termed LOCa [41]) will be used in the future to reversibly mediate Ca^2+^ influx in aneuploidy and further demonstrate that the survival of aneuploidy can be affected by Ca^2+^ homeostasis. Further understanding of the regulatory mechanism of genomic imbalance will provide an important scientific basis for studying the molecular mechanisms of human diseases.

## 5. Conclusion

In this study, the gene expression patterns of aneuploid *Drosophila* offspring from parents with unbalanced genomes were analyzed through reciprocal crosses, and the potential functions of the MSL2 protein in this process were studied. It was found that the ectopic expression of MSL2 in aneuploid offspring normalized gene expression patterns, aligning them more closely with diploidy. Notably, *ERp60,* a key target of MSL2, was identified as a critical regulator of ER Ca^2+^ homeostasis through its interaction with STIM1. The overexpression of ERp60 led to increased ER Ca^2+^ levels and improved survival rates in aneuploid females. In conclusion, this research underscores the critical role of MSL2 and ERp60 in the regulation of aneuploid *Drosophila*.

## Data Availability

The sequencing data have been deposited in the Gene Expression Omnibus (GEO) database (accessed on 17 May 2023) (https://www.ncbi.nlm.nih.gov/geo/, accession no. GSE162951).

## Supplementary Materials

The following supporting information can be downloaded at: https://www.mdpi.com/article/10.3390/cells13221923/s1, Figure S1: Genetic reciprocal crosses to generate aneuploidy *Drosophila*; Figure S2: Immunofluorescence of *Drosophila* polytene chromosomes from third instar larvae of trisomy 2L+ and MSL-trisomy 2L+; Figure S3: Immunofluorescence of *Drosophila* polytene chromosomes from third instar larvae of trisomy 2L- and MSL-trisomy 2L-; Figure S4: Relative fluorescence intensity of H4K16Ac in trisomy 2L; Figure S5: TSA-FISH of candidate genes in group 1; Figure S6: TSA-FISH of candidate genes in group 2; Figure S7: TSA-FISH of candidate genes in group 3; Figure S8: The ratio of expression quantity in three stages; Figure S9: TSA-FISH of TE Transpac in embryo; Figure S10: TSA-FISH of TE 3S18 in embryo; Figure S11: TSA-FISH of TE MAX in embryo; Figure S12: Over-expression of Hsc70-4 and ERp60 significantly improves the sex ratio of trisomy 2L; Figures S13–S15: Relative fluorescence intensity of RNA signal in trisomy 2L and MSL2-trisomy 2L obtained with reciprocal crosses compared with normal diploidy; Table S1: Primers used in RT-PCR; Table S2: Primers used in TSA-FISH.
