# Supplementary material for: Calcium Homeostasis Is Involved in the Modulation of Gene Expression by MSL2 in Imbalanced Genomes
Source: Cells. 2024 Nov 20;13(22):1923. doi: 10.3390/cells13221923 (PMC11593054; doi:10.3390/cells13221923)
Supplement: Supplementary file 1 [file cells-13-01923-s001.zip › cells-3215209-supplementary.pdf]

Supplementary materials

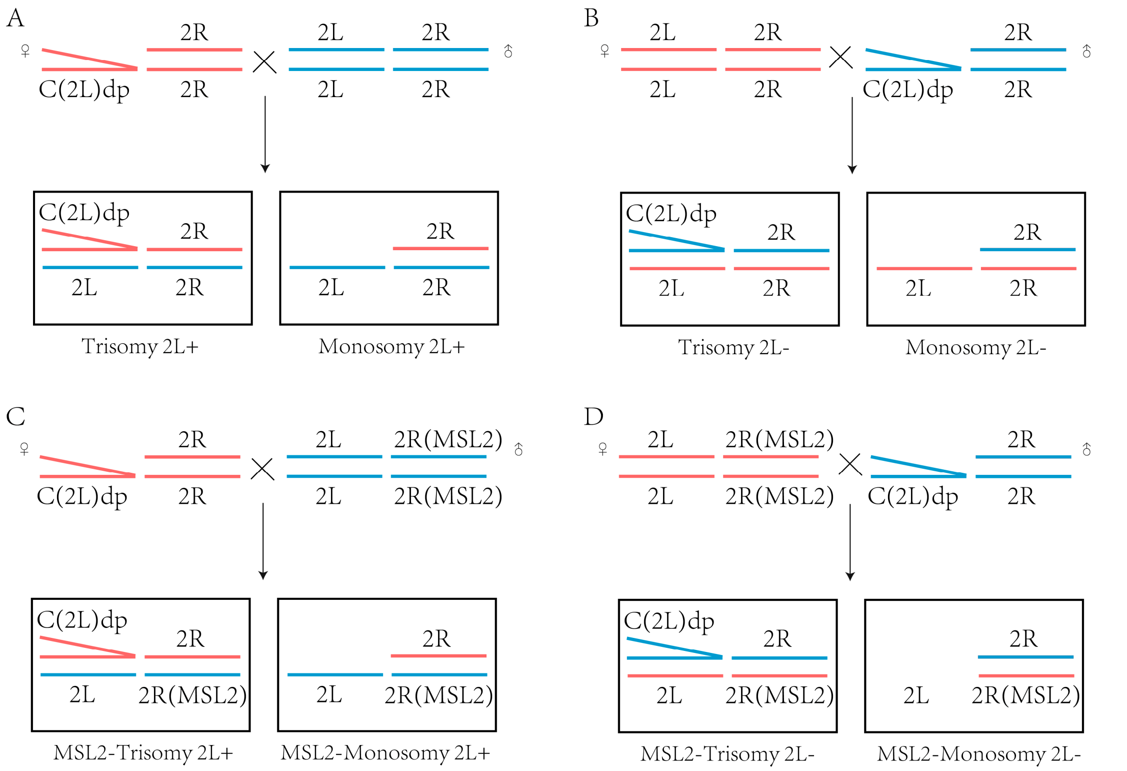

Figure S1. Genetic reciprocal crosses to generate aneuploidy *Drosophila*. (A) Cross of y; C(2L)dp; F(2R) bw females with Canton S males to produce trisomy 2L larvae, denoted as Trisomy 2L+. (B) Cross of Canton S females with y; C(2L)dp; F(2R) bw males to produce trisomy 2L larvae, denoted as Trisomy 2L-. (C) Cross of y; C(2L)dp; F(2R) bw females with MSL2/MSL2 males to produce trisomy 2L larvae, denoted as MSL2-trisomy 2L+. (D) Cross of MSL2/MSL2 females with y; C(2L)dp; F(2R) bw males to produce trisomy 2L larvae, denoted as MSL2-trisomy 2L-. (A-D) Only trisomy larvae can survive to the third instar stage.

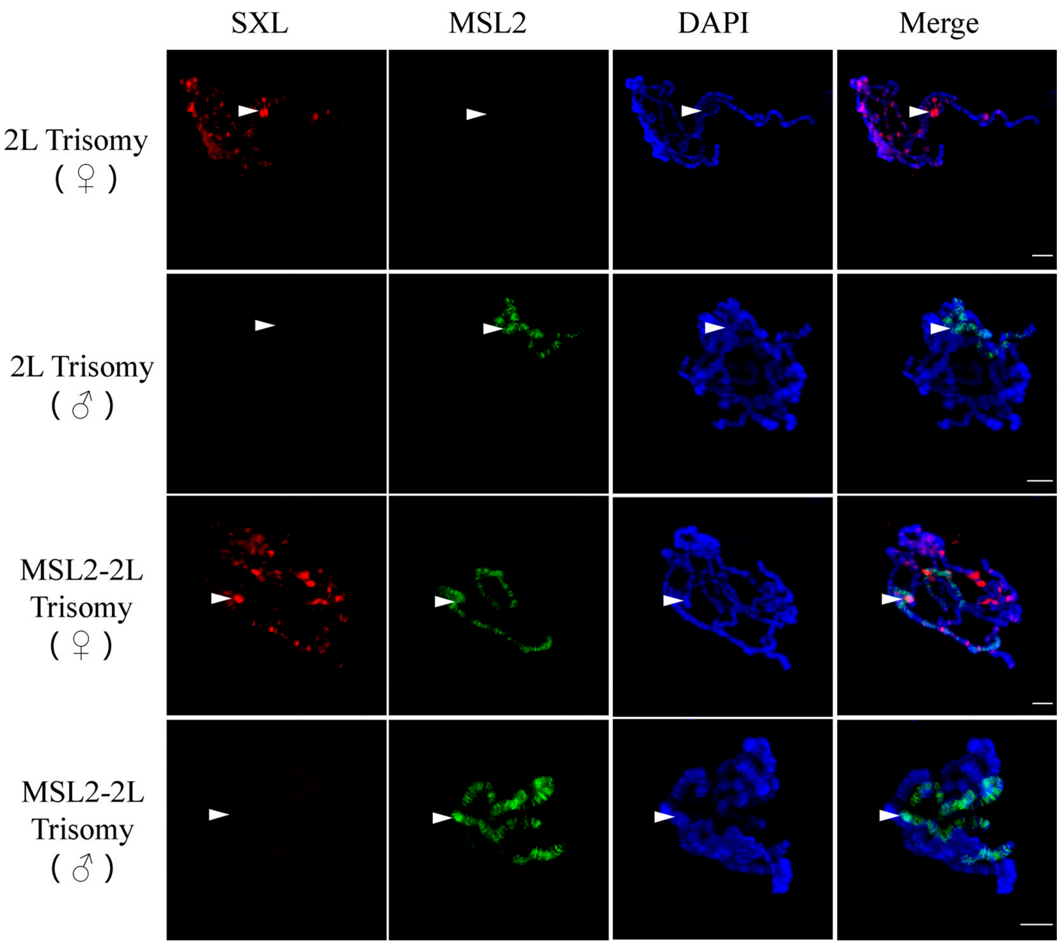

Figure S2. Immunofluorescence of *Drosophila* polytene chromosomes from third instar larvae of trisomy 2L+ and MSL-trisomy 2L+. The red channel is the signal from SXL and the green channel is the signal from MSL2. DNA is stained with DAPI in blue. Scale bars, 5 µm. SXL protein is only present in females and can be used to verify the sex of larvae. Arrowheads point to the enrichment of signals in the polytene chromosomes.

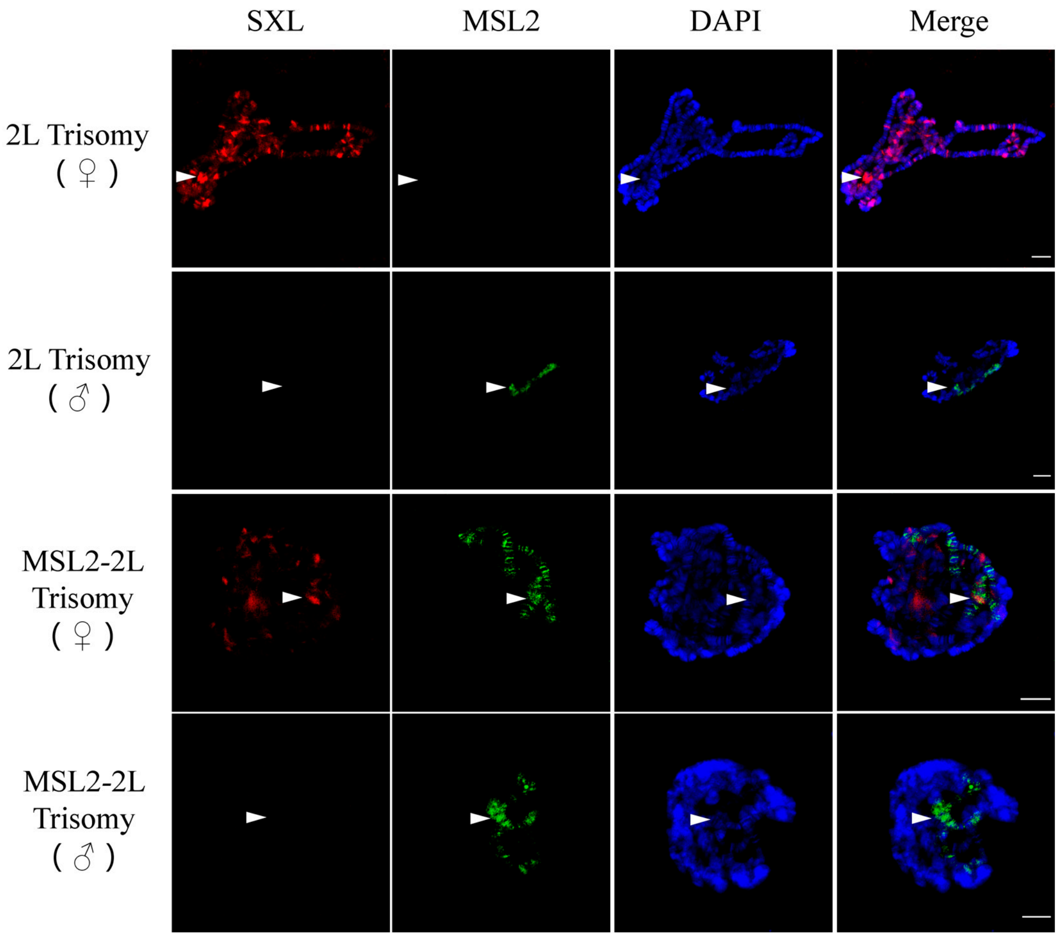

Figure S3. Immunofluorescence of *Drosophila* polytene chromosomes from third instar larvae of trisomy 2L- and MSL-trisomy 2L-. The red channel is the signal from SXL and the green channel is the signal from MSL2. DNA is stained with DAPI in blue. Scale bars, 5 µm. SXL protein is only present in females and can be used to verify the sex of larvae. Arrowheads point to the enrichment of signals in the polytene chromosomes.

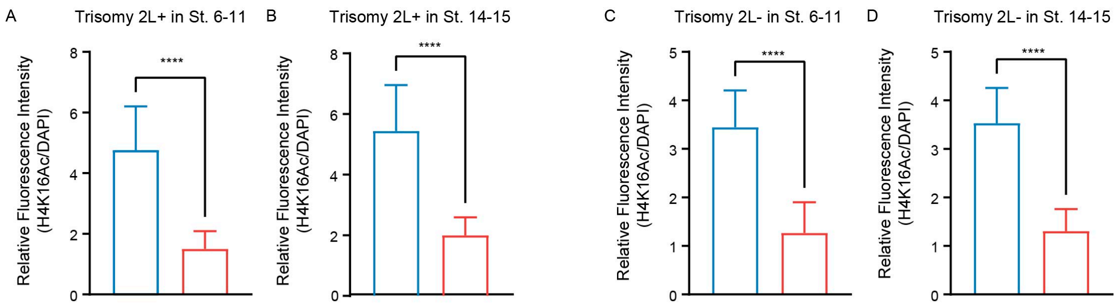

Figure S4. Relative fluorescence intensity of H4K16Ac in trisomy 2L. (A, B) Relative fluorescence intensity of H4K16Ac in trisomy 2L+ at stages 6–11 (A) and stages 14–15 (B). (C, D) Relative fluorescence intensity of H4K16Ac in trisomy 2L- at stages 6–11 (C) and stages 14–15 (D). The blue bars represent males, the red bars representfemales. The relative fluorescence intensity is determined by comparison with the DAPI signal. ****$p < 0.0001$.

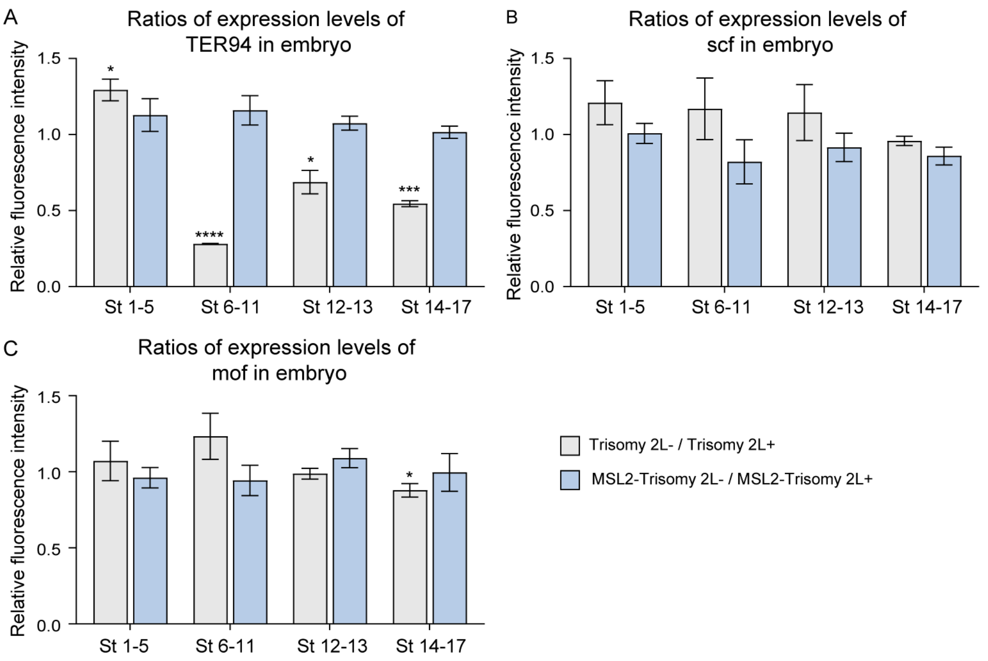

Figure S5. TSA-FISH of candidate genes in group 1. (A–C) The ratios of *TER94* expression levels (A), *scf* expression levels (B) and *mof* expression levels (C) of trisomy 2L- to trisomy 2L+ and MSL2-trisomy 2L- to MSL2-trisomy 2L+. The relative fluorescence intensity was calculated by the ratio of RNA signal to DAPI signal. Error bars indicate mean ± S.D. The One-Sample Wilcoxon Test was used to analyze statistical variance and the hypothesis value was set to 1. *p < 0.05; ***p < 0.001; ***p < 0.001; non-significant is omitted.

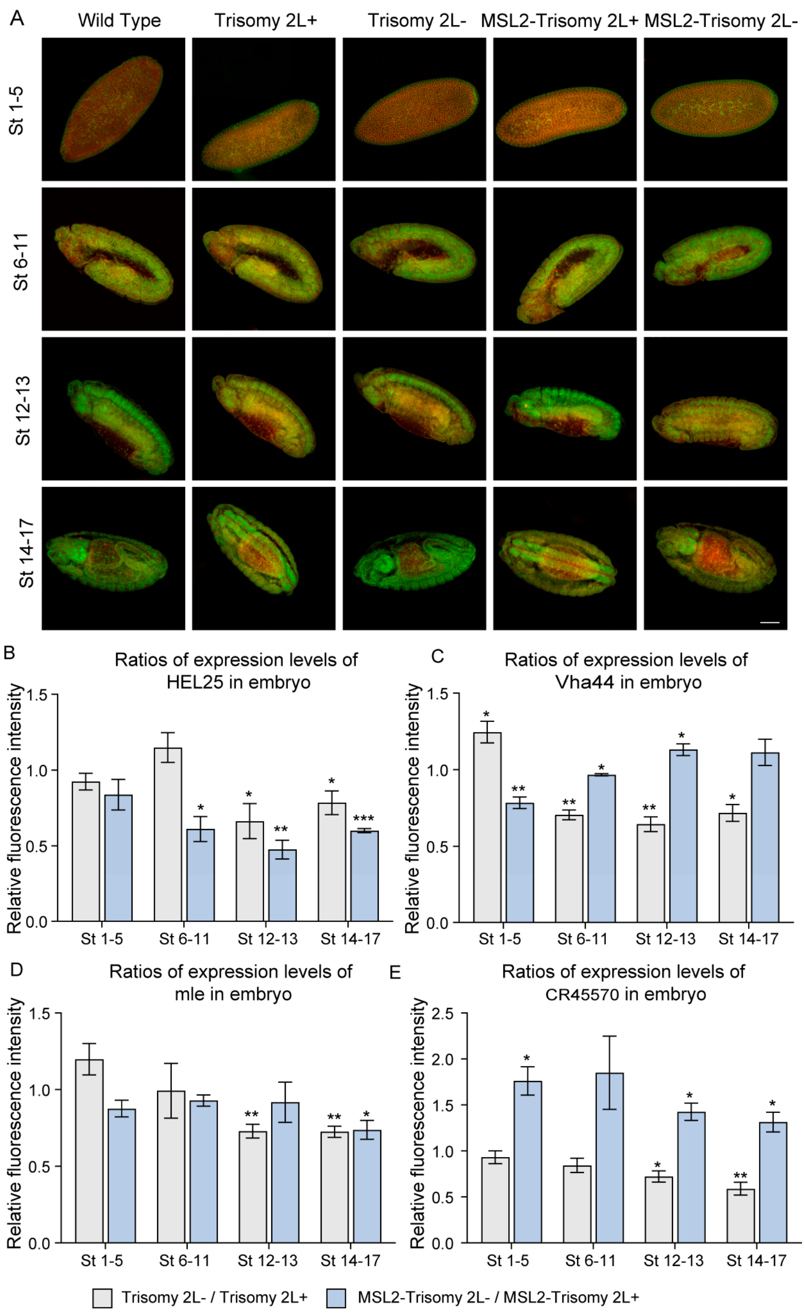

Figure S6. TSA-FISH of candidate genes in group 2. (A) Expression patterns of *Hel25E* in entire embryo. The genotype of the sample is shown above, and the development stage of the sample is shown on the left. The red pseudo-color is the signal from probe of *HEL25* and the green pseudo-color is the signal from nucleus. Scale bar, 80 µm. (B–E) The ratios of *Hel25E* expression levels (B), *Vha44* expression levels (C), *mle* expression levels (D) and *CR45570* expression levels (E) of trisomy 2L- to trisomy 2L+ and MSL2-trisomy 2L- to MSL2-trisomy 2L+. The relative fluorescence intensity was calculated as the ratio of RNA signal to DAPI signal. Error bars indicate mean ± S.D. The One-Sample Wilcoxon Test was used to analyze statistical variance and the hypothesis value was set to 1. *$p < 0.05$; **$p < 0.01$; ***$p < 0.001$; non-significant is omitted.

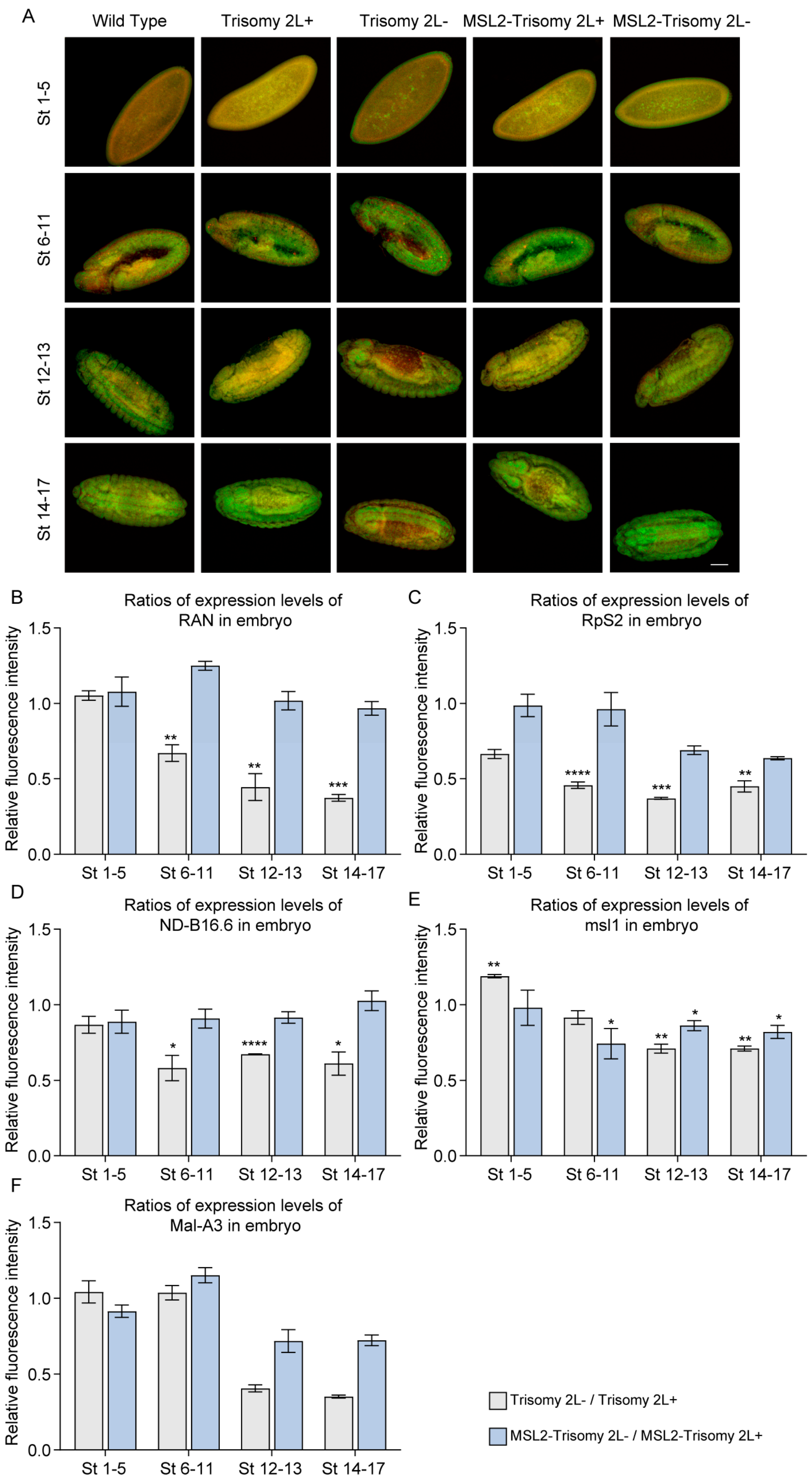

Figure S7. TSA-FISH of candidate genes in group 3. (A) Expression patterns of *Ran* in entire embryo. The genotype of the sample is shown above, and the development stage of the sample is shown on the left. The red pseudo-color is the signal from probe of *Ran* and the green pseudo-color is the signal from nucleus. Scale bars, 80 µm. (B–F) The ratios of *Ran*

expression levels (B), *RpS2* expression levels (C), *ND-B16.6* expression levels(D), *msl-1* expression levels (E) and *Mal-A3* expression levels (F) of trisomy 2L- to trisomy 2L+ and MSL2-trisomy 2L- to MSL2-trisomy 2L+. The relative fluorescence intensity was calculated asthe ratio of RNA signal to DAPI signal. Error bars indicate mean ± S.D. The One-Sample Wilcoxon Test was used to analyze statistical variance and the hypothesis value was set to 1. *$p < 0.05$; **$p < 0.01$; ***$p < 0.001$; ****$p < 0.0001$; non-significant is omitted.

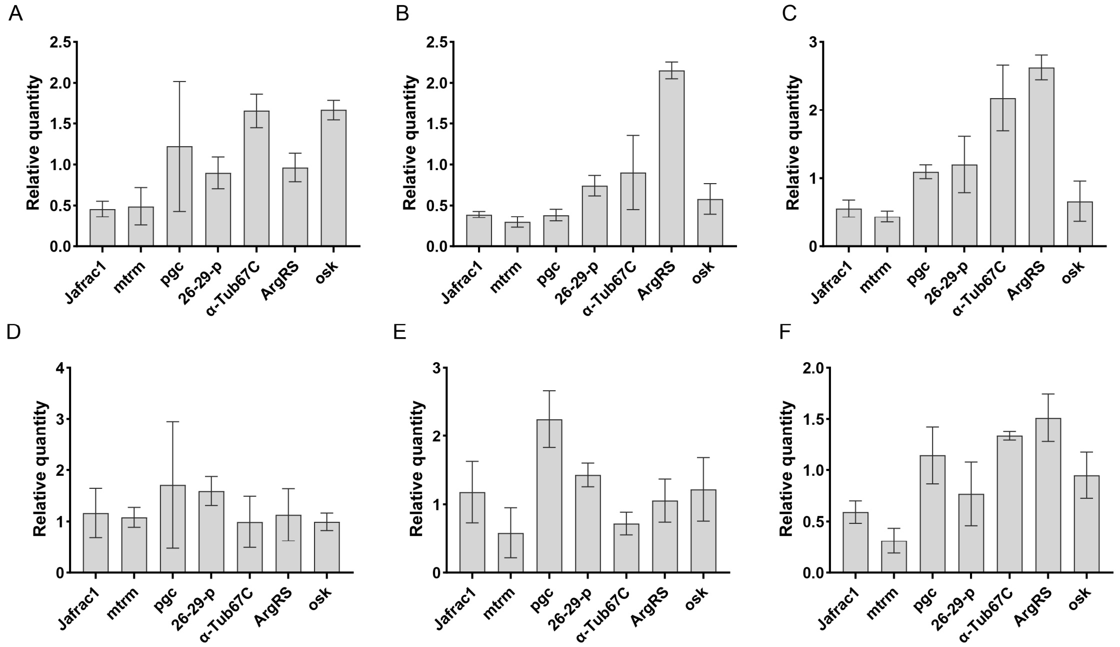

Figure S8. The ratio of expression quantity in three stages. (A–C) The ratio of expression quantity between trisomy 2L obtained by reciprocal crosses in pre-MZT and pre-ZGA (A), post-ZGA (B) and post-MZT (C). (D–F) The ratio of expression quantity between MSL2-trisomy 2L obtained by reciprocal crosses in pre-MZT and pre-ZGA (D), post-ZGA (D), and post-MZT (F). The two-tailed Student's t test was used to analyze statistical variance. Data expressed as means of 3 independent experiments. Error bars indicate mean ± S.D.

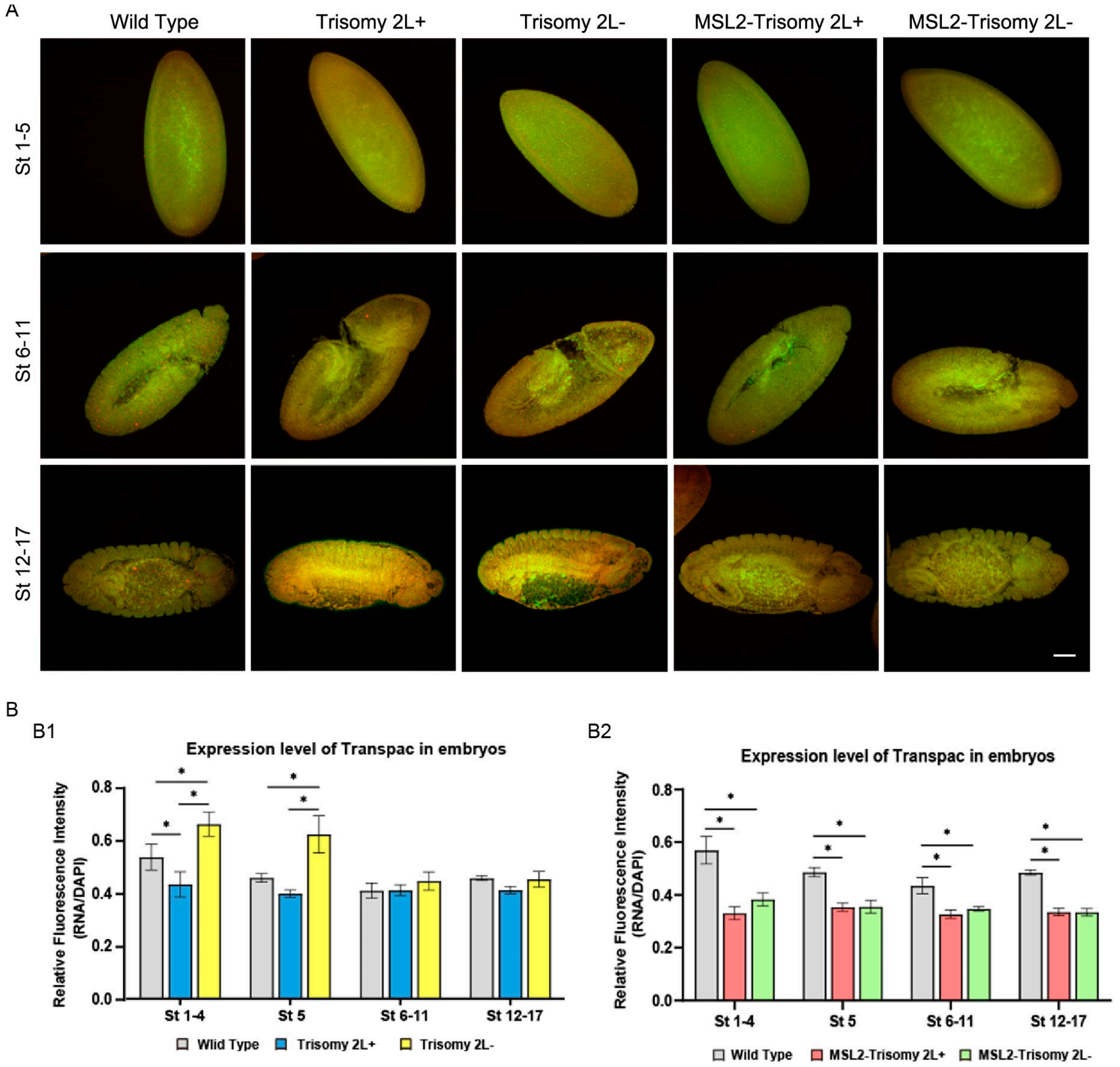

Figure S9. TSA-FISH of TE Transpac in embryo. (A) TE Transpac expression patterns in entire embryo. The genotype of the sample is shown above, and the development stages of the sample is shown on the left. Green is the DNA signal, and red is the RNA probe signal. The result shown in the figure is the merge of red and green fluorescence. Scale bar, 63 μm. (B) Relative fluorescence intensity of Transpac signal in trisomy 2L and MSL2-trisomy 2L compared with normal diploidy. The relative fluorescence intensity was calculated as the ratio of the RNA signal to the DAPI signal. (B1) Relative fluorescence intensity of Transpac signal in wild type and trisomy 2L contained by reciprocal crosses. (B2) Relative fluorescence intensity of Transpac signal in wild type and MSL2-trisomy 2L contained by reciprocal crosses. Asterisk denotes a p-value < 0.05 by two-tailed Student’s t tests.

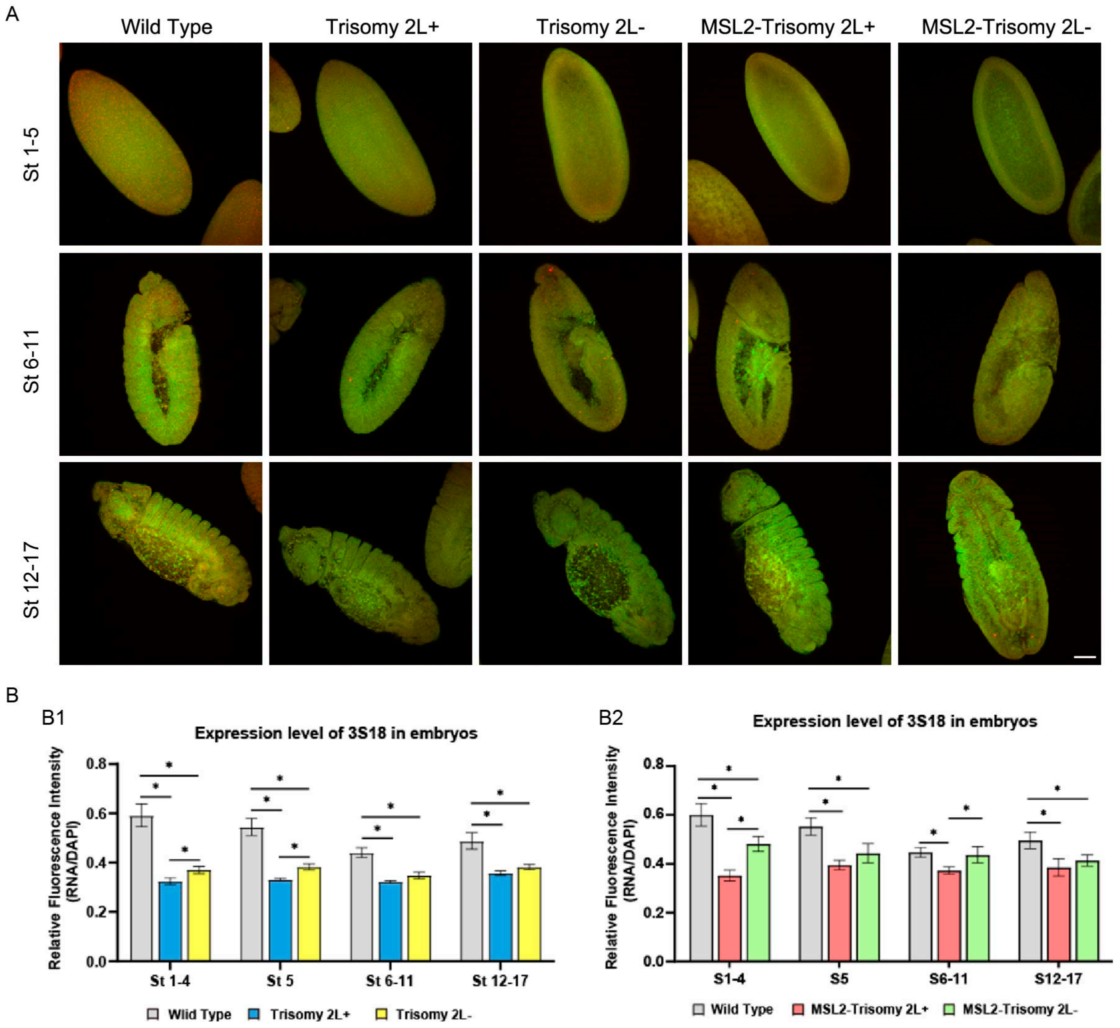

Figure S10.TSA-FISH of TE 3S18 in embryo. (A) TE 3S18 expression patterns in entire embryo. The genotype of the sample is shown above, and the development stages of the sample is shown on the left. Green is the DNA signal, and red is the RNA probe signal. The result shown in the figure is the merge of red and green fluorescence. Scale bar, 63 µm. (B) Relative fluorescence intensity of 3S18 signal in trisomy 2L and MSL2-trisomy 2L compared with normal diploidy. The relative fluorescence intensity was calculated as the ratio of the RNA signal to the DAPI signal. (B1) Relative fluorescence intensity of 3S18 signal in wild type and trisomy 2L contained by reciprocal crosses. (B2) Relative fluorescence intensity of 3S18 signal in wild type and MSL2-trisomy 2L contained by reciprocal crosses. Asterisk denotes a p value < 0.05 by two-tailed Student's t tests.

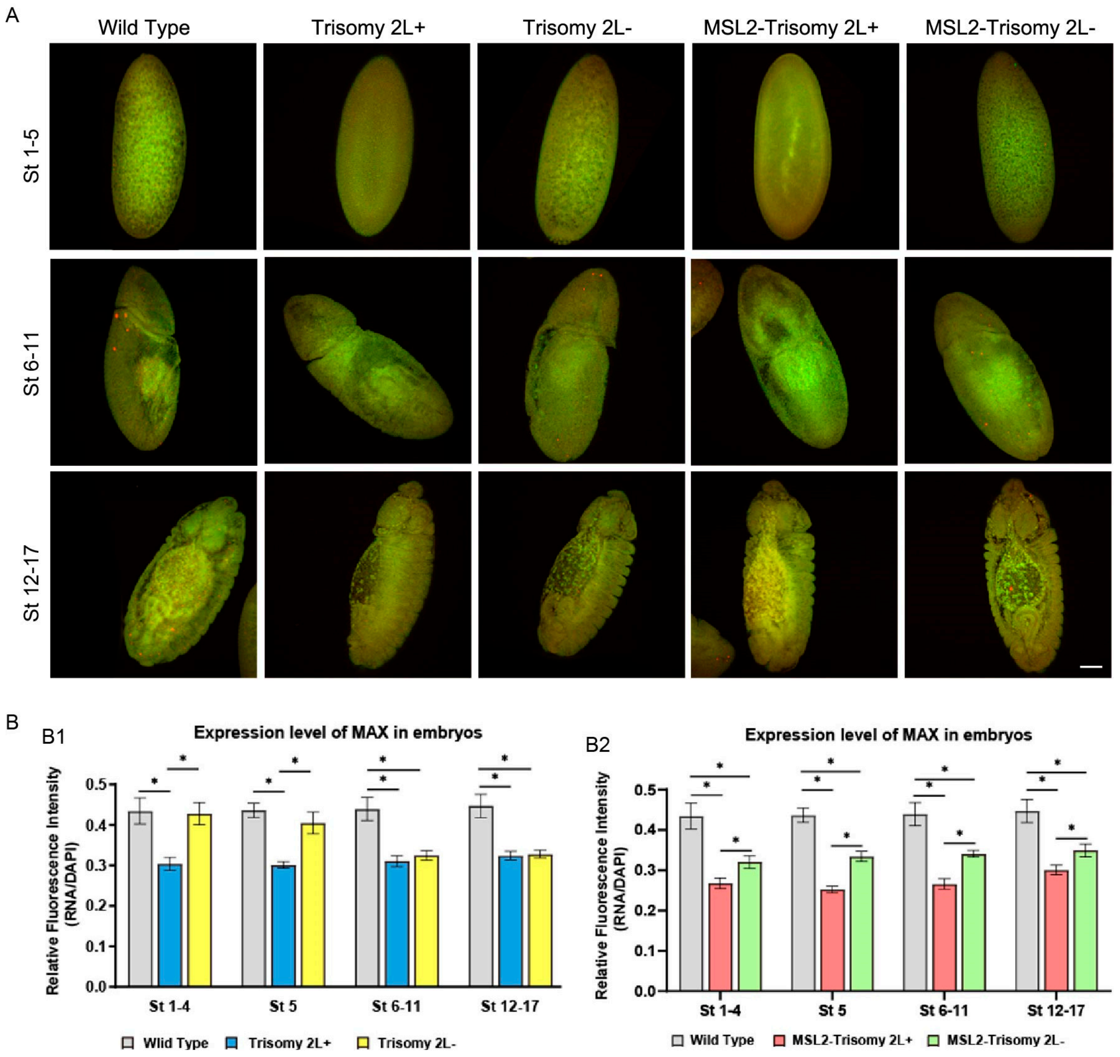

Figure S11.TSA-FISH of TE MAX in embryo. (A) TE MAX expression patterns in entire embryo. The genotype of the sample is shown above, and the development stages of the sample is shown on the left. Green is the DNA signal, and red is the RNA probe signal. The result shown in the figure is the merge of red and green fluorescence. Scale bars, 63 μm. (B) Relative fluorescence intensity of MAX signal in trisomy 2L and MSL2-trisomy 2L compared with normal diploidy. The relative fluorescence intensity was calculated as the ratio of the RNA signal to the DAPI signal. (B1) Relative fluorescence intensity of MAX signal in wild type and trisomy 2L contained by reciprocal crosses. (B2) Relative fluorescence intensity of MAX signal in wild type and MSL2-trisomy 2L contained by reciprocal crosses. Asterisk denotes a p value < 0.05 by two-tailed Student’s t tests.

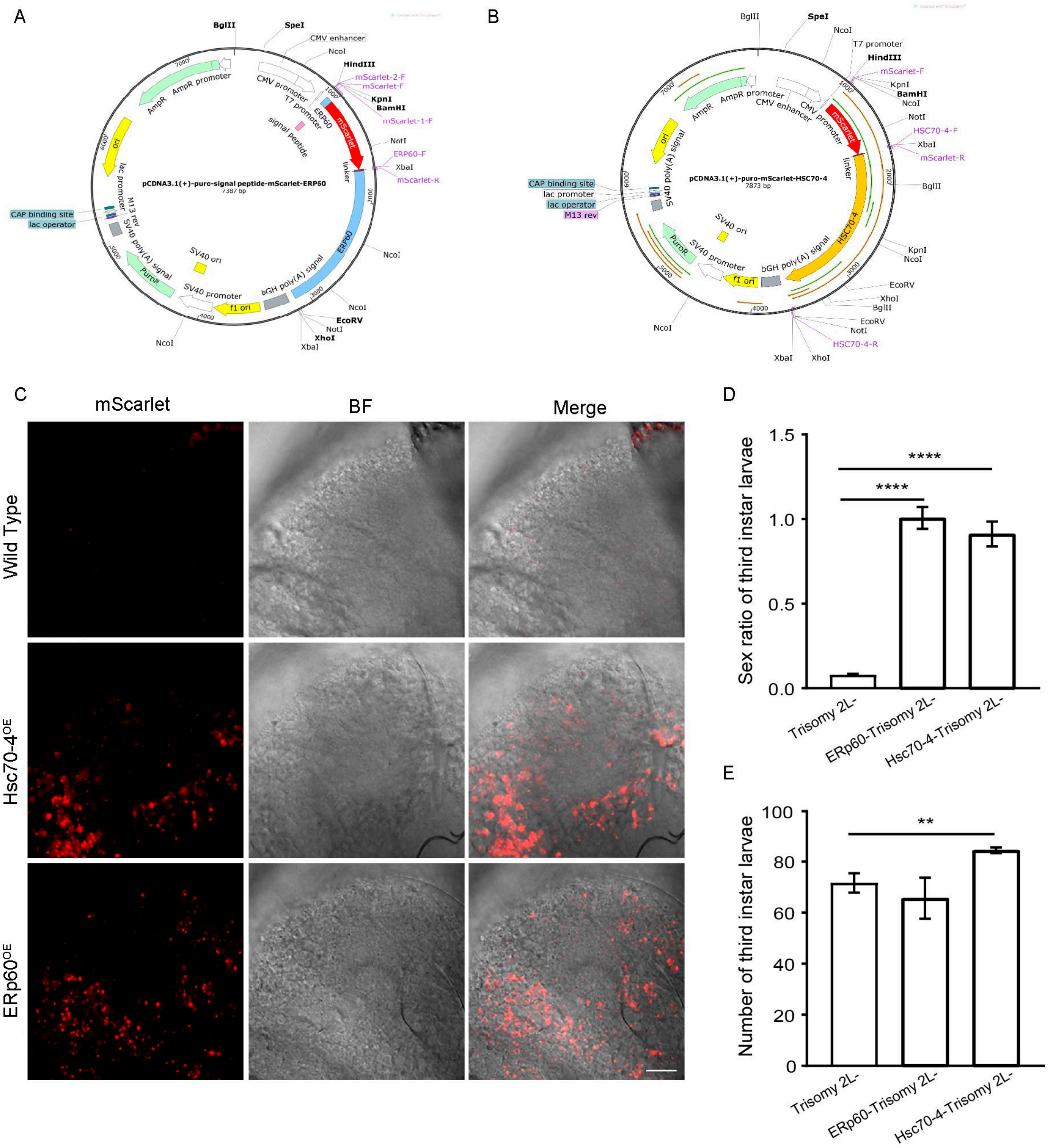

Figure S12. Overexpression of Hsc70-4 and ERp60 significantly improves the sex ratio of trisomy 2L. (A, B) Plasmid profile. The sequence of mScarlet was first amplified by PCR from PEB2-mScarlet and then subcloned into the pcDNA3.1(+) backbone by using a multiple fragment homologous recombination kit. (A) Plasmid profile of pcDNA3.1(+)-puro-mScarlet-ERp60. The signal peptide encoding the sequence of ERp60 and the remaining nucleotides were inserted upstream and downstream of the mScarlet gene, correspondingly. (B) Plasmid profile of pcDNA3.1(+)-puro-mScarlet-Hsc70-4. The nucleotides of Hsc70-4 were inserted upstream of the mScarlet gene. (C) The red fluorescence signal of ERp60 and Hsc70-4 in brains. mScarlet, red fluorescent protein. Scale bar, 20 μm. (D, E) The sex ratio (female to male) (A) and the number (B) of trisomy2L-, ERp60-trisomy 2L-, and Hsc70-4-trisomy 2L-. Data expressed as means of 3 independent experiments. Error bars indicate mean ± S.D. $^{**}p<0.01$, $^{****}p<0.0001$.

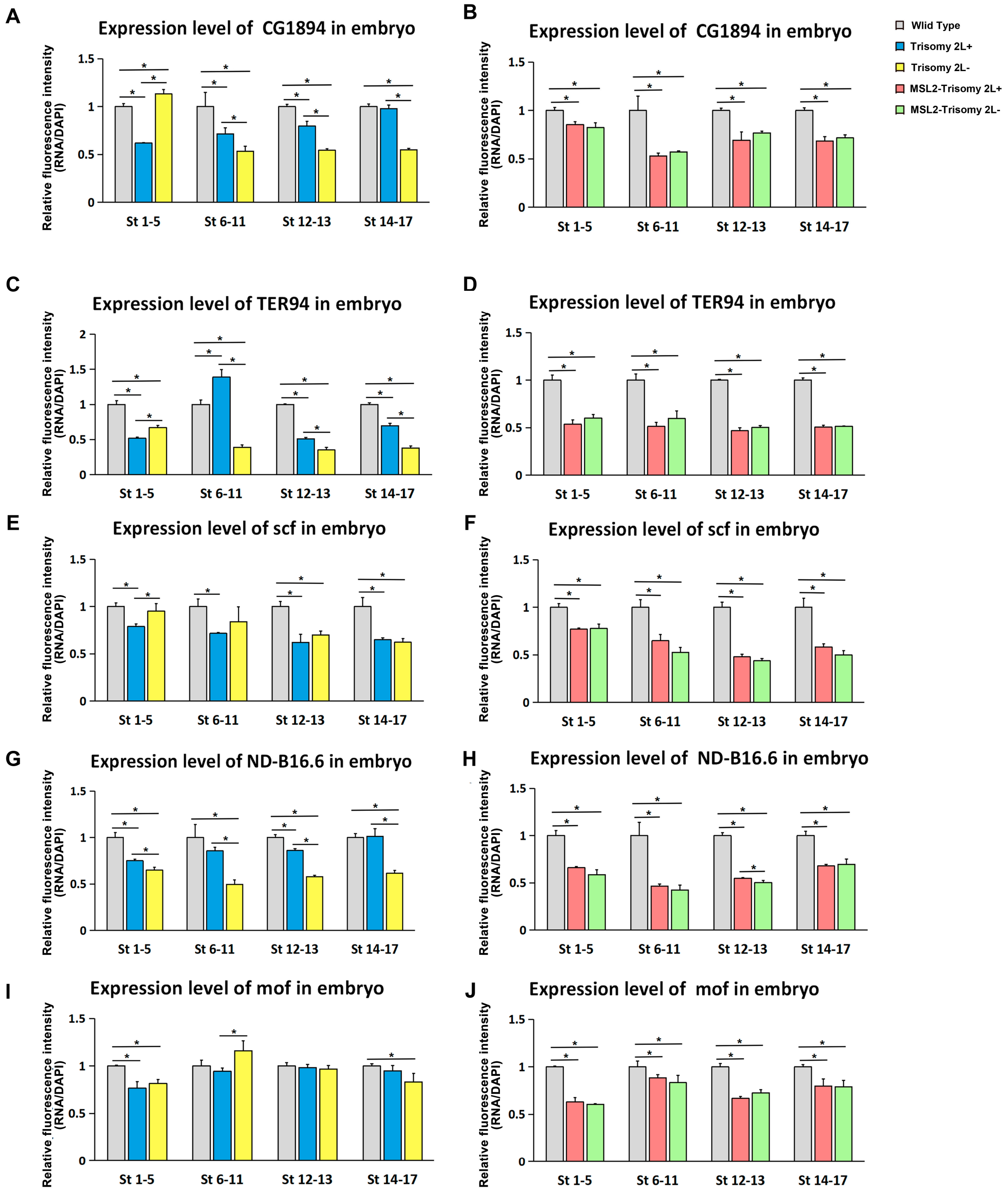

Figure S13. Relative fluorescence intensity of RNA signal in trisomy 2L and MSL2-trisomy 2L obtained with reciprocal crosses compared with normal diploidy, representing candidate genes CG1894 (A, B), TER94 (C, D), scf (E, F), ND-B16.6 (G, H), mof (I, J) respectively. The relative fluorescence intensity was calculated as the ratio of the RNA signal to the DAPI signal. The expression of wild-type embryos at each stage of development are set to 1. Asterisk denotes a p value < 0.05 obtained via two-tailed Student's t test.

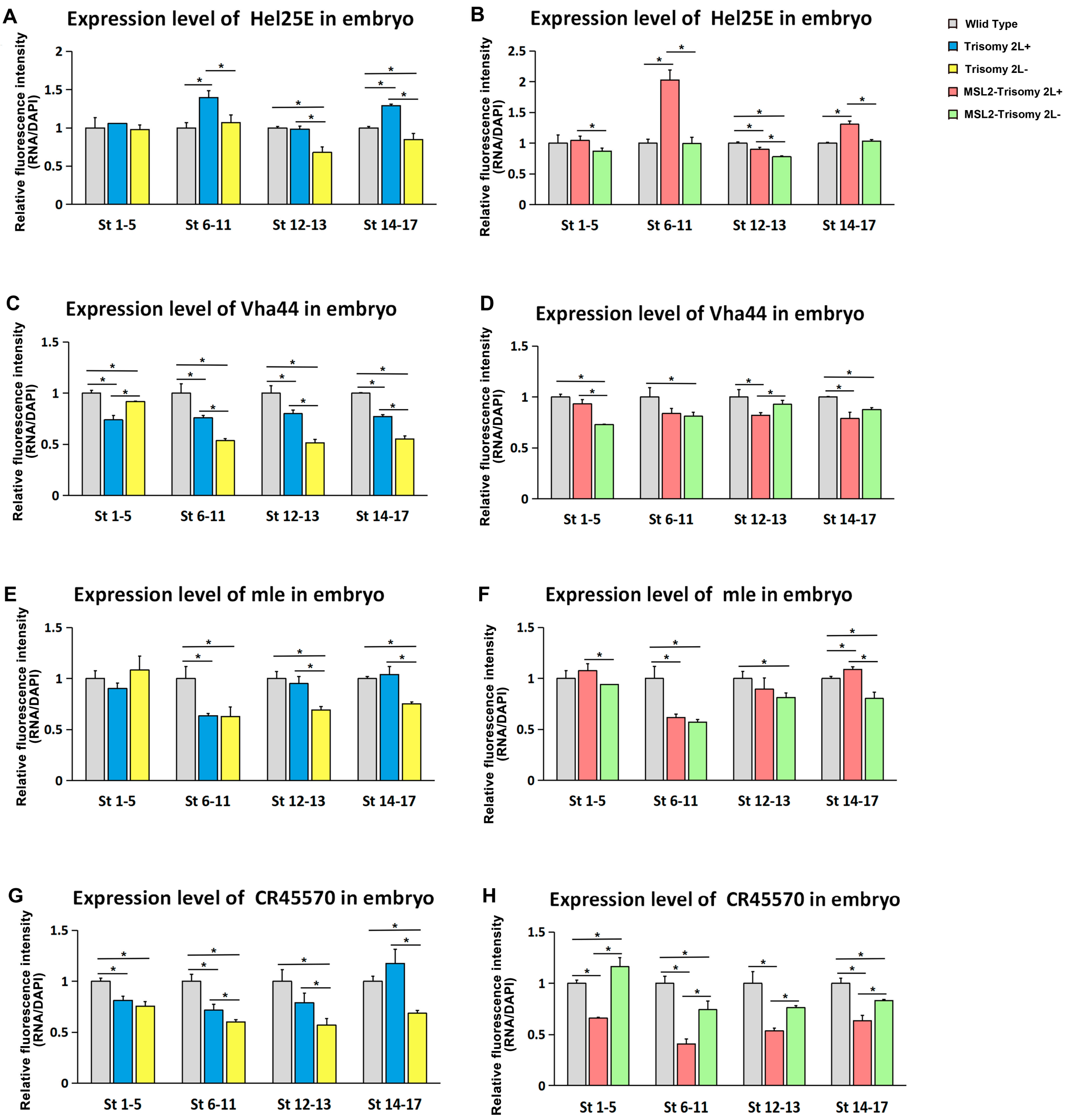

Figure S14. Relative fluorescence intensity of RNA signal in trisomy 2L and MSL2-trisomy 2L obtained with reciprocal crosses compared with normal diploidy, representing candidate genes Hel25E (A, B), Vha44 (C, D), mle (E, F), CR45570 (G, H) respectively. The relative fluorescence intensity was calculated as the ratio of the RNA signal to the DAPI signal. The expression of wild-type embryos at each stage of development are set to 1. Asterisk denotes a p value < 0.05 obtained via two-tailed Student's t test..

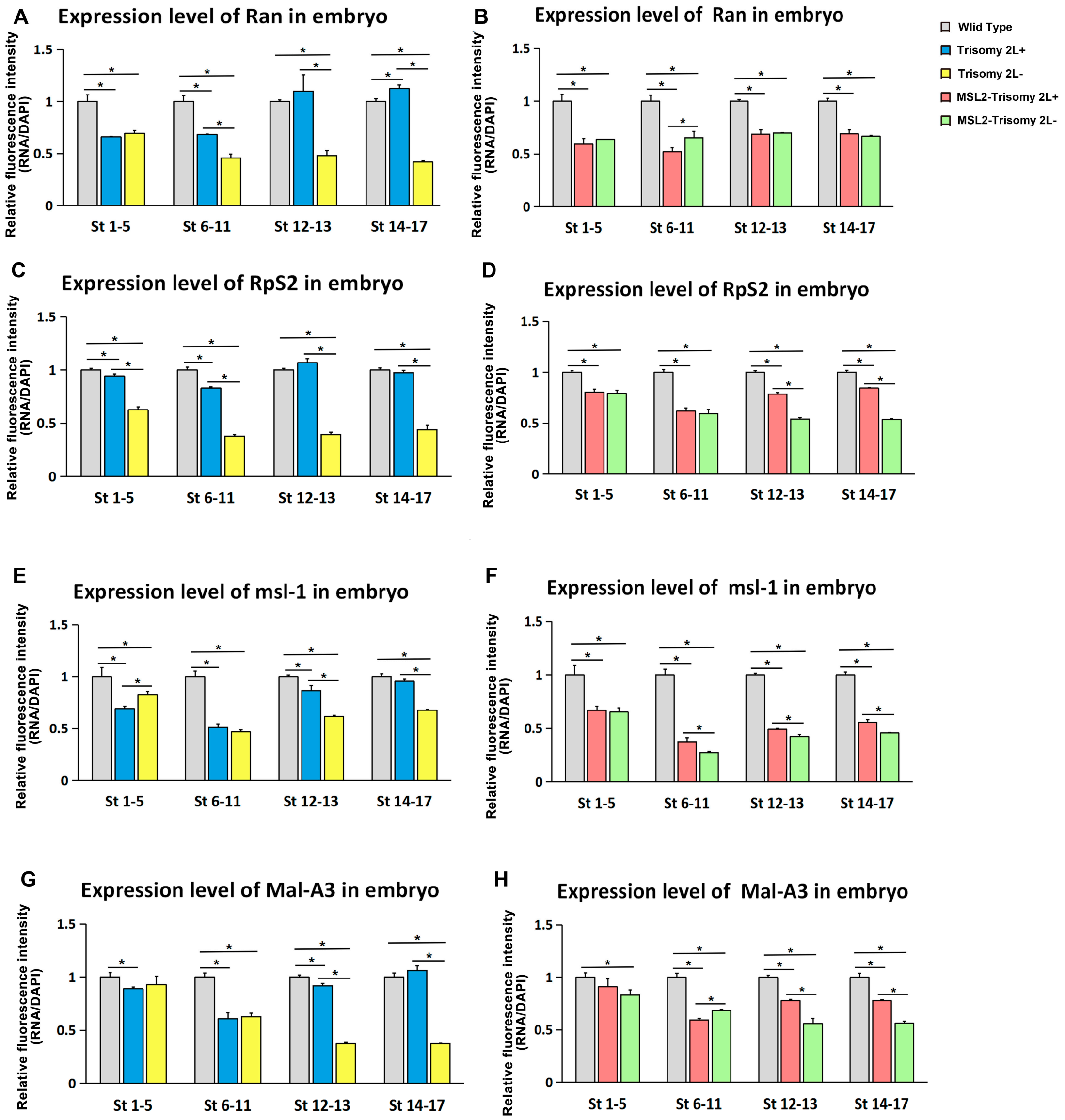

Figure S15. Relative fluorescence intensity of RNA signal in trisomy 2L and MSL2-trisomy 2L obtained with reciprocal crosses compared with normal diploidy, representing candidate genes Ran(A, B), RpS2 (C, D), msl-1 (E, F), Mal-A3 (G, H) respectively. The relative fluorescence intensity was calculated as the ratio of the RNA signal to the DAPI signal. The expression of wild-type embryos at each stage of development are set to 1. Asterisk denotes a p value < 0.05 obtained via two-tailed Student's t test.

Table S1. Primers used in RT-PCR.

Genes	Left primer	Right primer
β-tubulin	AGCTCAGCACCCTCTGTGTAAT	AGCTGGAGCGCATCAATGTGTA
Jafrac1	CTGGTGCTTTTCTTCTACCC	CACCTTCATCGACTTGTCAG
26-29-p	GAACATCTATACGCTGTGGG	CTCGTAGCTGTCATAGTCCA
mtrm	CCGGAGCGAGAAATAAAGAG	TGGTCACATAGTTCTCCAGG
pgc	TAACTACCAGCCTTCAGAGG	CGAGCTTCCCTACTTGTTCT
α-Tub67C	CATCAACTACGAGAAGCCAG	GTTCCAGTACGGCGATATTC
OSK	GTCAGGACTTTGTGTATCCG	CTCCTTGATCAGTAGCCGAA
ArgRS-m	CGGAACACATAGTGGTTGAG	GTACAGCGTTTCTATTGGGG

Table S2. Primers used in TSA-FISH.

Types	Transcripts	Left primer	Right primer
mRNA	Hel25E	TTCACATCGAAACGATCCTG	TTGTGAAGTCTGTGCAACG
	RpS2	CATATGTAGCCTTGGCGAAG	ATCTACCTGTACTCGCTTCC
	msl-1	GCATCCCTACACTTTCGATC	CCCGTCTGATATTCGGAAAC
	mle	AAGGGCGAGACCATAGATAG	CAGTAATCGAAGCTGAGGTG
	Mal-A3	CTATTCCACGCTCCTTCTTC	GATCTAAACTACCGCAACCC
	TER94	CTTTTGACCCTGATGGATGG	TTAGCCTTCAGGATAGCCTC
	ERp60	TAAGGAAGTCGTCTACCTCG	ACTACCAGAAGAACCCCAAG
	Vha44	GGCTTATTGAACCACCAGTC	GATCTCAAGGGTAAATCCGC
	scf	CACCAAGGATGAGTTTACCG	CGAAAACGTCGTACTTGTCT
	Hsc70-4	GGTGCTTACCAAGATGAAGG	CATCGGGATTGATCGACTTG
	CG1894	CTGAGGTAACAAAGTCTGGC	CGAAGCTCAAAGAATAGGGG
	CR45570	CACCGCTAGAAATTGGACTC	AACGAGGATTGGAGTAGGAG
	roX1	GTTGGACCGGATTTCATAGG	CTACACCCAGAAGAAACTGC
	mof	GGCCCTTCCAATACTTGATC	GTCCTATTGCGAGAACAGTC
	ND-B16.6	GGGCGTAGAACTCCTTGAAA	GCCTGGGCATCTACTACTTG
	Ran	CACAGAGGACGATTGGTATG	GCTCAGGAAGGTCAGGATAT
Transposable element	Transpac	GTTTGCCAGAGAGGAGATTG	AAACTAGGGGTCTATGGTCG
	3S18	GGTAACGACTTTCCTATCGC	GAGCTGTCTTTTCCTGATCC
	Max	TACGAGTGAAGAGATCCGAG	GGATAGCCTTCGTGGAAAAG
	flea	GTCGGTAAAAGTCGGGAAAG	AGTCGTGTAGAGTTTCCTCC

The sequences of promoters connected to the flanks of the primers are omitted. The forward primers connect T3 promoter: TGTTGGGAAATCACTCCCAATTAA and the reverse primers connect T7 promoter: GTAATACGACTCACTATAGGGAGACCAC.
